# The Inactivation of Enzymes Belonging to the Central Carbon Metabolism Is a Novel Mechanism of Developing Antibiotic Resistance

**DOI:** 10.1128/mSystems.00282-20

**Published:** 2020-06-02

**Authors:** Teresa Gil-Gil, Fernando Corona, José Luis Martínez, Alejandra Bernardini

**Affiliations:** a Centro Nacional de Biotecnología, CSIC, Madrid, Spain; Marquette University

**Keywords:** fosfomycin, antibiotic resistance, central carbon metabolism, Embden-Meyerhof-Parnas pathway, *Stenotrophomonas maltophilia*

## Abstract

Fosfomycin is a bactericidal antibiotic, analogous to phosphoenolpyruvate, that exerts its activity by inhibiting the activity of MurA. This enzyme catalyzes the first step of peptidoglycan biosynthesis, the transfer of enolpyruvate from phosphoenolpyruvate to uridine-diphosphate-*N*-acetylglucosamine. Fosfomycin is increasingly being used, mainly for treating infections caused by Gram-negative multidrug-resistant bacteria. The mechanisms of mutational resistance to fosfomycin in Stenotrophomonas maltophilia, an opportunistic pathogen characterized by its low susceptibility to commonly used antibiotics, were studied in the current work. None of the mechanisms reported so far for other organisms, which include the production of fosfomycin-inactivating enzymes, target modification, induction of an alternative peptidoglycan biosynthesis pathway, and the impaired entry of the antibiotic, are involved in the acquisition of such resistance by this bacterial species. Instead, the unique cause of resistance in the mutants studied is the mutational inactivation of different enzymes belonging to the Embden-Meyerhof-Parnas central metabolism pathway. The amount of intracellular fosfomycin accumulation did not change in any of these mutants, showing that neither inactivation nor transport of the antibiotic is involved. Transcriptomic analysis also showed that the mutants did not present changes in the expression level of putative alternative peptidoglycan biosynthesis pathway genes or any related enzyme. Finally, the mutants did not present an increased phosphoenolpyruvate concentration that might compete with fosfomycin for its binding to MurA. On the basis of these results, we describe a completely novel mechanism of antibiotic resistance based on mutations of genes encoding metabolic enzymes.

**IMPORTANCE** Antibiotic resistance has been largely considered a specific bacterial response to an antibiotic challenge. Indeed, its study has been mainly concentrated on mechanisms that affect the antibiotics (mutations in transporters, efflux pumps, and antibiotic-modifying enzymes, or their regulators) or their targets (i.e., target mutations, protection, or bypass). Usually, antibiotic resistance-associated metabolic changes were considered a consequence (fitness costs) and not a cause of antibiotic resistance. Herein, we show that alterations in the central carbon bacterial metabolism can also be the cause of antibiotic resistance. In the study presented here, Stenotrophomonas maltophilia acquires fosfomycin resistance through the inactivation of glycolytic enzymes belonging to the Embden-Meyerhof-Parnas pathway. Besides resistance to fosfomycin, this inactivation also impairs the bacterial gluconeogenic pathway. Together with previous work showing that antibiotic resistance can be under metabolic control, our results provide evidence that antibiotic resistance is intertwined with the bacterial metabolism.

## INTRODUCTION

Antibiotic resistance (AR) can be considered a chemical problem. To be active, an antibiotic requires reaching its target at concentrations high enough to inhibit its activity. Any process or situation that either reduces the effective concentration of the antibiotic or the antibiotic target affinity should lead to AR. In agreement with this situation, classical mechanisms of resistance described so far (1) include elements that diminish the antibiotic concentration like efflux pumps (2), antibiotic-inactivating enzymes (3), or changes in the antibiotic transporters (4). Concerning the target, elements that reduce its affinity with the antibiotic include mutations (5), target protection (6), bypass (7) or replacement (8) and eventually increased target expression (9). Studies of the intrinsic resistome have shown that, in addition to these classical resistance determinants, the susceptibility to antibiotics of a bacterial species depends on the activity of several elements encompassing all functional categories (10–12). However, little is still known about the interplay between bacterial metabolism and the acquisition of AR (13). In the current article, we explore this feature analyzing Stenotrophomonas maltophilia fosfomycin-resistant mutants. Fosfomycin is a phosphonic acid derivative that contains an epoxide and a propyl group. It is chemically analogous to phosphoenolpyruvate (PEP), which explains its mechanism of action (14). The enzyme MurA (UDP-*N*-acetylglucosamine enolpyruvyl transferase), which catalyzes the first step in peptidoglycan biosynthesis (15), the transfer of enolpyruvate from PEP to uridine diphospho-*N*-acetylglucosamine, is the only known fosfomycin target. Fosfomycin binds covalently to a cysteine residue in the active site of MurA, which renders MurA inactive. As a consequence of MurA inactivation, the peptidoglycan precursor monomers accumulate inside the cell, peptidoglycan cannot be synthesized, and this leads to bacterial cell lysis and death (16).

Different molecular mechanisms leading to fosfomycin resistance have been identified (17), some of which impair fosfomycin/MurA interaction. Some allelic variants of MurA found in pathogens intrinsically resistant to fosfomycin such as Mycobacterium tuberculosis, Borrelia burgdorferi, or *Chlamydia* sp. (15, 18–20) do not contain a cysteine in their active site, and therefore, they are not fully inhibited by fosfomycin. In the case of organisms containing a fosfomycin-sensitive MurA allele, mutations in *murA* have been selected in the laboratory (15, 21, 22). In addition, it has been shown that the increased synthesis of MurA also confers a fosfomycin resistance phenotype (23, 24). Also, the presence of an alternative route of peptidoglycan synthesis, as it happens in Pseudomonas putida and Pseudomonas aeruginosa, may allow circumventing the activity of fosfomycin by recycling the peptidoglycan without the need for *de novo* synthesis by the MurA enzyme (7). Regarding mechanisms that involve a reduction in the intracellular concentration of the antibiotic, resistance can be achieved as the consequence of changes in the entry of fosfomycin inside a bacterial cell. The main cause of this impaired uptake is the selection of mutations in any of the genes encoding the sugar phosphate transporters GlpT and UhpT, which are the gates for fosfomycin entry (25, 26). Note here that expression of these transporters is under metabolic control, in such a way that situations where the nutritional bacterial status favors the use of sugar phosphates (as intracellular growth) increase fosfomycin activity (27, 28). Finally, in other cases, fosfomycin is inactivated by fosfomycin-modifying enzymes such as FosA, FosB, and FosX (29–32). All the mechanisms of fosfomycin resistance already known fit in the classical categories of resistance elements (see above). However, the results presented in the current article suggest that none of them is involved in the acquisition of resistance by S. maltophilia. In this bacterial species, fosfomycin resistance was acquired due to mutations in genes encoding enzymes of the Embden-Meyerhof-Parnas (EMP) metabolic pathway. It has been suggested that AR can be interlinked to bacterial metabolism (33, 34). However, with very few exceptions (35), the mutational inactivation of genes encoding enzymes involved in central carbon metabolism has not been considered a significant cause of AR in bacterial pathogens (34, 35). Our article hence shed light on the cross talk between AR and central carbon metabolism in S. maltophilia.

## RESULTS

### Selection of S. maltophilia fosfomycin-resistant mutants and identification of the mutations involved.

In order to isolate single-step S. maltophilia fosfomycin-resistant mutants, around 10^8^ bacterial cells were seeded on selection plates containing fosfomycin (1,024 μg/ml). Four single-step fosfomycin-resistant mutants, hereafter dubbed FOS1, FOS4, FOS7, and FOS8, were selected for further studies. The MICs to fosfomycin of the mutants were determined. In all cases, the fosfomycin MICs were higher in the mutants than in the wild-type strain (Table 1), with the MICs of the four mutants higher than 1,024 μg/ml. We have also measured the fosfomycin MICs of a set of clinical S. maltophilia isolates (36). Unlike the mutants selected *in vitro*, none of these isolates had a fosfomycin MIC higher than 384 μg/ml (Table 2), indicating that it is unlikely that they possess the mutations described here. Predicting mutations that might be involved in the acquisition of antibiotic resistance for antibiotics currently in use or for novel antibiotics to be introduced in clinics is fundamental to tackling the problem of antibiotic resistance (37, 38). Consequently, the study of above-described mutants is a first approach that will provide information on potential fosfomycin resistance mechanisms in S. maltophilia even before they are used in clinics.

**TABLE 1:** MICs of fosfomycin for the resistant mutants and their corresponding complemented strains

Strain	Fosfomycin MIC (μg/ml) for strain with the following plasmid used for complementation^*a*^:					
	None	pSEVA234	pSEVA234*eno*	pSEVA234*gpmA*	pSEVA234*gapA*	pSEVA234*pgk*
D457	192	192	128	128	128	192
FOS1	>1,024	>1,024	192	ND	ND	ND
FOS4	>1,024	>1,024	ND	192	ND	ND
FOS7	>1,024	>1,024	ND	ND	128	ND
FOS8	>1,024	>1,024	ND	ND	ND	192

a ND, not done (each strain was complemented with the wild-type allele of the corresponding mutated gene).

**TABLE 2:** MICs of fosfomycin for S. maltophilia clinical isolates

Isolate	Origin	Fosfomycin MIC (μg/ml)
D457		192
E729	Urine	96
E227	Blood	96
E759	Sputum	384
E999	Respiratory secretion	256
G51	Blood	48
E539	Pus from a wound	88
E301	Urine	112
D388	Urine	96
C048	Urine	96
E861	Sputum	48
C357	Urine	40
F375	Blood	320
E824	Blood	80

The genomes of the FOS mutants were fully sequenced and compared with that of the parental wild-type strain D457. Five different mutations were detected. FOS4, FOS7, and FOS8 carried one mutation, while FOS1 harbored two mutations. One of these mutations (in *rne*, SMD_RS14705:c.G1464T:p.E488D) was discarded because it was predicted to be neutral using the Provean predictor (0.41 score) and because as shown below, the complementation of the other mutation present in this strain allows recovery of its susceptibility to fosfomycin to the level of the wild-type strain, indicating that the *rne* mutation does not participate in this phenotype. Notably, each mutant contains a different mutation, but all four were found in genes encoding enzymes of the EMP metabolic pathway, namely, *eno*, *gpmA*, *gapA*, and *pgk* (Table 3). The presence of the mutations was confirmed in all cases by PCR amplification and subsequent Sanger sequencing of the amplicons.

**TABLE 3:** SNPs mapped in the mutants with low susceptibility to fosfomycin

Mutant	Position of the reference sequence	SNP	Locus	Gene	Amino acid change^*a*^	Product	Old locus tag	Provean score^*b*^	SNP domain
FOS1	1829461	A:T>G:C	SMD_RS08765	*eno*	p.D398G	Enolase	SMD_1655	−6.77	C-terminal TIM barrel domain
FOS4	1411119	C:G>T:A	SMD_RS06650	*gpmA*	p.P212L	Phosphoglycerate mutase	SMD_1268	−9.88	Histidine phosphatase superfamily
FOS7	3798418	G:C>C:G	SMD_RS17680	*gapA*	p.D296G	Glyceraldehyde-3- phosphate dehydrogenase	SMD_3406	−3.37	NAD binding domain
FOS8	3793349	G:C>A:T	SMD_RS17665	*pgk*	p.Q50ST	Phosphoglycerate kinase	SMD_3403		N-terminal domain, containing the substrate binding site

a ST, stop mutation.

b Provean deleterious score threshold of the changes is −2.5. Any SNP is located in a catalytic site of the corresponding enzyme.

Although no other mutations seemed to be the cause of the resistance of the mutants studied, the wild-type allele of the corresponding mutated gene was introduced into each mutant strain to obtain functional validation of the effects of these mutations in the susceptibility to fosfomycin of S. maltophilia. As shown in Table 1, introduction of the wild-type forms of such genes fully restored the susceptibility of the analyzed S. maltophilia fosfomycin-resistant mutants to the level of the wild-type strain. These results indicate that the fosfomycin resistance of these mutants is solely due to the mutation of genes encoding enzymes of the EMP metabolic pathway. It may be possible that the observed resistance could be due to an improved general stress response of the mutants. To address this possibility, the susceptibility to other antibiotics was tested in the fosfomycin-resistant mutants. No relevant general changes in the susceptibility to the tested antibiotics were found between the wild-type strain and the mutants (Table 4). However, mutant-specific MIC changes of two- or threefold compared to the parental strain were observed for some antibiotics. These results strongly suggest that the resistance mechanisms caused by these mutations in genes encoding enzymes involved in central metabolism are fosfomycin specific.

**TABLE 4:** Susceptibility to different antibiotics of mutants with low susceptibility to fosfomycin

Antibiotic	MIC (μg/ml) of the following strain to the indicated antibiotic:				
	D457	FOS1	FOS4	FOS7	FOS8
Gentamicin	3	4	6	3	4
Tobramycin	2	3	4	3	3
Ciprofloxacin	1.5	2	3	2	3
Nalidixic acid	12	12	12	32	24
Ceftazidime	1	1	1	2	1
Colistin	4	4	2	6	8
Tetracycline	1.5	1.5	0.75	1	1
Chloramphenicol	3	6	4	6	4

To the best of our knowledge, there are no reports about S. maltophilia clinical isolates either presenting high-level fosfomycin resistance in general or possessing these single nucleotide polymorphisms (SNPs) in the mutated genes. Further, we did not detect any strain presenting MICs for fosfomycin as high as those of the selected mutants (Table 2) from a collection of clinical S. maltophilia isolates. Nevertheless, it may be possible that these mutations could be found on available S. maltophilia genomes. To explore this possibility, the sequences of *eno*, *gpmA*, *gapA*, and *pgk* genes from 39 clinical isolates from different sources, present in the NCBI database (39) were compared to the sequences of the wild-type D457 strain. All sequences were highly conserved, and none of the sequences has any of the nucleotide replacements found in the FOS mutants (see Fig. S1 in the supplemental material). To further analyze potential changes in the enzymes involved in fosfomycin resistance, *eno*, *gpmA*, *gapA*, and *pgk* sequences of the 39 genomes were translated to protein and compared to the protein sequences of the wild-type D457 strain and the corresponding FOS mutant. None of the genomes presented the same amino acid replacement as the ones observed in the FOS mutants (Fig. S2).

10.1128/mSystems.00282-20.1 FIG S1 Analysis of SNPs in clinical S. maltophilia isolates. (A) Enolase. (B) Phosphoglycerate mutase A. (C) Glyceraldehyde-3-phosphate dehydrogenase A. (D) Phosphoglycerate kinase. Thirty-nine clinical isolates from different clinical origins were selected for the screening. The SNPs involved in the acquisition of resistance in our study are highlighted with a square. SNPs in FOS1, FOS4, FOS7, and FOS8 are shown in Table 3. *eno* A:T > G:C; *gpmA* C:G > T:A; *gapA* G:C > C:G; *pgk* G:C > A:T. Mutations analogous to those found in FOS1, FOS4, FOS7, and FOS8 mutants were not detected in any of the clinical isolates. Download FIG S1, PDF file, 1.9 MB. Copyright © 2020 Gil-Gil et al. 2020 Gil-Gil et al. This content is distributed under the terms of the Creative Commons Attribution 4.0 International license.

10.1128/mSystems.00282-20.2 FIG S2 Analysis of amino acid changes in clinical S. maltophilia isolates. (A) Enolase. (B) Phosphoglycerate mutase. (C) Glyceraldehyde-3-phosphate dehydrogenase. (D) Phosphoglycerate kinase. Thirty-nine clinical isolates from different clinical origins were selected for the screening. The amino acid changes involved in the acquisition of resistance in our study are highlighted with a square. The positions of the sequences of the wild-type strain D457 and the mutants are shown with arrows. Amino acid changes in FOS1, FOS4, FOS7, and FOS8 are shown in Table 3. Changes analogous to those found in FOS1, FOS4, FOS7, and FOS8 mutants were not detected in any of the clinical isolates. Download FIG S2, PDF file, 1.5 MB. Copyright © 2020 Gil-Gil et al. 2020 Gil-Gil et al. This content is distributed under the terms of the Creative Commons Attribution 4.0 International license.

### Model of S. maltophilia central metabolism.

**FIG 1:**
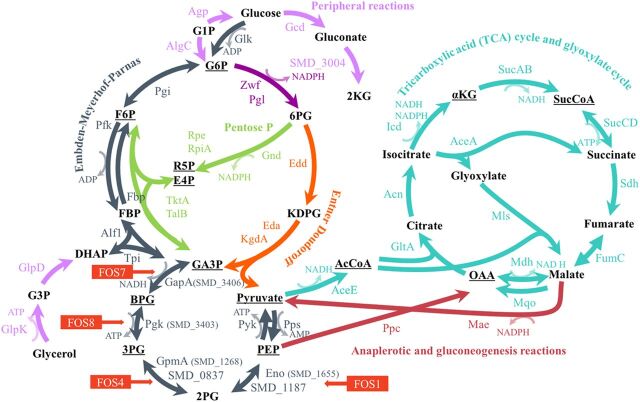
Central metabolism of S. maltophilia D457. Schematic representation of the main pathways of the central metabolism: glycolysis (Entner-Doudoroff and Embden-Meyerhof-Parnas), tricarboxylic acid cycle, and glyoxylate cycle, pentose phosphate pathway, and anaplerotic and gluconeogenic reactions, as well as peripheral reactions. Underlined are the essential precursors for the biomass formation (40). The mutated enzyme in each FOS mutant is indicated with the name of the corresponding mutant. The abbreviations and names of substrates, products, and enzymes, as well as their locus tags, are shown in the legend to Fig. S3 in the supplemental material.

As a first step for deciphering how the mutations in genes encoding enzymes of the EMP metabolic pathway may impact S. maltophilia physiology, a metabolic map of the central metabolism, which generates energy and precursors to form biomass (40), was modeled for S. maltophilia. The EMP pathway is the best-analyzed glycolytic route. It is based on the sequential activity of 10 individual enzymes. The first five form the upper glycolytic pathway (Glk, Pgi, Pfk, Alf1, and TpiA) in which, using ATP, hexoses are converted into triose phosphate, whereas in the lower glycolytic pathway (GapA, Pgk, GpmA, Eno, and PykA), pyruvate is formed from the triose phosphate, at the same time that NADH and ATP are generated. The pyruvate obtained is decarboxylated by the action of the pyruvate dehydrogenase complex and enters as acetyl coenzyme A (acetyl-CoA) to the tricarboxylic acid (TCA) cycle (41). The EMP pathway may also function in a gluconeogenic regime, forming hexose phosphates from triose phosphates (42). The loci of all enzymes of the EMP pathway were identified in S. maltophilia D457 (Fig. 1 and Fig. S3). Moreover, the Entner-Doudoroff (ED) route, another glycolytic pathway that also forms triose phosphates from hexose phosphates, is present as well in S. maltophilia. It is important to notice that two enzymes of the central metabolism of strain D457, GpmA and Eno, present isoenzymes capable of carrying out the same chemical reaction. As shown in Fig. 1, all the fosfomycin resistance mutations are located in genes encoding enzymes of the lower glycolytic pathway.

10.1128/mSystems.00282-20.3 FIG S3 Supplemental information for Fig. 1. (A) Central metabolism of S. maltophilia D457. The substrate abbreviations and enzymes (short form and enzyme names) follow. Substrate abbreviations: G1P, glucose-1-phosphate (glucose-1P); G6P, glucose-6P; F6P, fructose-6P; FBP, fructose-1,6P; DHAP, dihydroxyacetone-P; G3P, glycerol-3P; GA3P, glyceraldehyde-3P; BPG, 1,3-bisphosphoglycerate; 3PG, 3P-glycerate; 2PG, 2P-glycerate; PEP, phosphoenolpyruvate; AcCoA, acetyl-coenzyme A; 2KG, 2-keto-gluconate; 6PG, 6P-gluconate; KDPG, 2-keto-3-deoxy-d-gluconate-6-phosphate; αKG, α-ketoglutarate; SucCoA, succinyl-coenzyme A; OAA, oxalacetate; E4P, erythrose-4P; R5P, ribulose-5P. Enzymes: Zwf, G6P 1-dehydrogenase; Pgl, 6-phosphogluconolactonase; for the Embden-Meyerhof-Parnas pathway, Glk, glucokinase; Pgi, G6P isomerase; Fbp, FBP phosphatase; Pfk, phosphofructokinase; Alf1, FBP aldolase; TpiA, triose-phosphate isomerase; GapA, GA3P dehydrogenase; Pgk, phosphoglycerate kinase; GpmA, phosphoglycerate mutase; Eno, enolase; Pyk, pyruvate kinase; Pps, phosphoenolpyruvate synthase; for the Entner-Doudoroff pathway, Edd, 6PG dehydratase; Eda/KgdA, KDPG aldolase; for the pentose phosphate pathway, Gnd, 6PG dehydrogenase; Rpe, pentose-5-phosphate-3-epimerase; RpiA, R5P isomerase A; TktA, transketolase; TalB, transaldolase; for the tricarboxylic acid cycle and glyoxylate cycle, AceE, pyruvate dehydrogenase; GltA, citrate synthase; Acn, aconitate hydratase; Icd, isocitrate dehydrogenase; SucAB, αKG dehydrogenase; SucCD, SucCoA synthase; Sdh, succinate dehydrogenase; FumC, fumarate hydratase; Mdh, malate dehydrogenase; Mqo, malate:quinone oxidoreductase; AceA, isocitrate lyase; Mls, malate synthase; for peripheral oxidation and phosphorylation of glucose reactions, Gcd, glucose dehydrogenase; Gad (SMD_3004), gluconate 2-dehydrogenase; Agp, glucose-1-phosphatase; AlgC, phosphomannomutase; GlpK, glycerol kinase; GlpD, G3P dehydrogenase; for peripheral oxidation and phosphorylation glucose reactions, Ppc, PEP carboxylase; Mae, malic enzyme. (B) Enzymes belonging to each pathway. Download FIG S3, JPG file, 0.5 MB. Copyright © 2020 Gil-Gil et al. 2020 Gil-Gil et al. This content is distributed under the terms of the Creative Commons Attribution 4.0 International license.

### Fosfomycin resistance mutations impair the activity of enzymes of the S. maltophilia central carbon metabolism.

**FIG 2:**
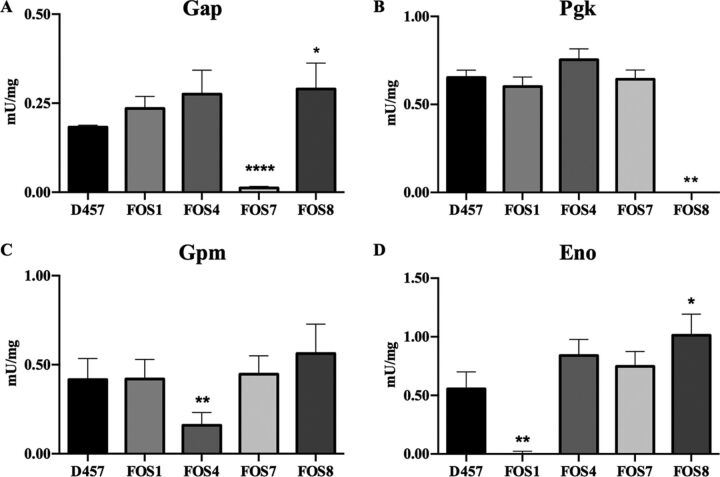
Enzymatic activity of the enzymes of the lower glycolytic pathway of the D457 parental strain and the fosfomycin-resistant mutants. (A) Gap, glyceraldehyde-3P dehydrogenase activity. (B) Pgk, phosphoglycerate kinase activity. (C) Gpm, phosphoglycerate mutase activity. (D) Eno, enolase activity. Error bars indicate standard deviations for the results from three independent replicates. As shown, each of the mutants exhibits an impaired activity of the enzyme encoded by the mutated gene. Values that are significantly different from the value for the wild-type D457 strain by a unpaired two-tail *t* test are indicated by asterisks as follows: *, *P* < 0.02; **, *P* < 0.002; ****, *P* < 0.0001.

To determine whether the mutations cause a loss of function of the encoded proteins, the enzymatic activity of Gap, Pgk, Gpm, and Eno was measured in the mutants and in the wild-type strain. As shown in Fig. 2, Gap activity decreased by 93% in the FOS7 mutant, Pgk activity decreased by 100% in FOS8, Gpm activity decreased by 65% in FOS4, and Eno activity decreased by 100% in FOS1 in relation to the parental strain. Thus, every mutation causes a loss of function of the gene.

**FIG 3:**
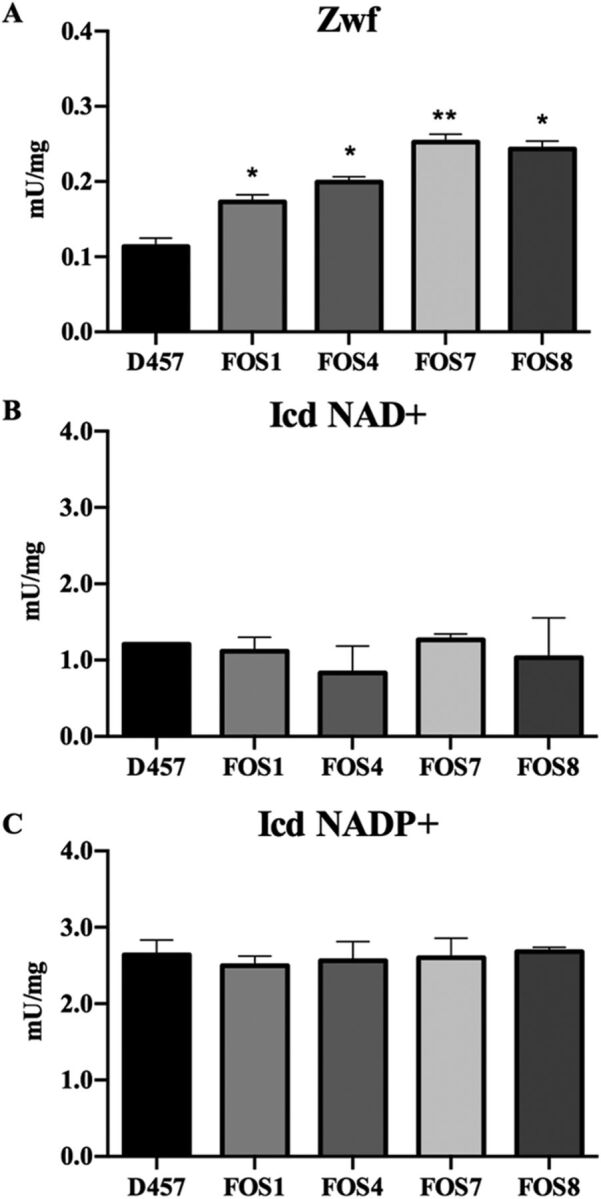
Enzymatic activity of dehydrogenases from S. maltophilia central metabolism of D457 parental strain and fosfomycin-resistant mutants. (A) Zwf glucose-6P dehydrogenase activity. (B) Icd (NAD+) isocitrate dehydrogenase NAD+ activity. (C) Icd (NADP+) isocitrate dehydrogenase NADP+ activity. Error bars indicate standard deviations for the results from three independent replicates. As shown, the activity of Zwf is higher in the fosfomycin-resistant mutants. Statistical significance calculated by unpaired two-tail *t* test: *, *P* < 0.02; **, *P* < 0.005.

To elucidate whether the reduced activity of these enzymes in the mutants may produce a relevant metabolic shift in S. maltophilia, the activities of the main dehydrogenases of the central metabolism of S. maltophilia D457, which are indicative of the general physiological state of the cell, including its redox balance, were measured. In particular, the activities of the glucose-6-phosphate (glucose-6P) dehydrogenase (Zwf), which connects the glucose-6P with the ED and pentose phosphate (PP) pathways, and the isocitrate dehydrogenases (Icd NAD^+^ and Icd NADP^+^), from the TCA cycle, were determined. The activity of the enzyme Zwf increased by 1.5- to 2.5-fold in the four fosfomycin-resistant mutants compared with the wild-type D457 strain, whereas the activities of either Icd NAD^+^ or Icd NADP^+^ enzymes did not change in any of the mutants studied (Fig. 3).

### Fosfomycin resistance is not the consequence of a metabolic rearrangement that modifies S. maltophilia susceptibility to oxidative stress.

It has been proposed that the activity of antibiotics may depend on the bacterial oxidative response (43). One of the key elements in this response is Zwf, an enzyme with a critical role in the supply of NADPH, which is a relevant cofactor for maintaining the cellular redox balance (44, 45). We have observed that this enzyme presented increased activity in the mutants compared with the wild-type strain (see above). To address whether this increased activity might be the reason for fosfomycin resistance, *zwf* was inactivated in the FOS4 and FOS7 mutants and in the wild-type D457 strain. The inactivation of *zwf* caused a slight increase in fosfomycin MIC levels from 192 to 256 μg/ml in the wild-type D457 strain, whereas this inactivation did not change fosfomycin susceptibility in the mutants tested.

In addition, the roles of the mutations in the response to oxidative stresses were tested by analyzing the susceptibility of the mutants to H_2_O_2_ and menadione. As shown in Table 5, the mutations conferring fosfomycin resistance did not alter the susceptibility of S. maltophilia to these compounds, whereas as expected, *zwf* inactivation caused an increase in the susceptibility to these oxidative stressors. These results indicate that the susceptibility to fosfomycin of S. maltophilia mutants with defective enzymes of the lower glycolytic pathway is a specific phenotype, not due to a change in the oxidative response.

**TABLE 5:** Susceptibility to oxidative stress of the mutants analyzed^*a*^

Strain	H_2_O_2_ (cm)	Menadione (cm)
D457	4.4 ± 0.2	1.9 ± 0.2
FOS1	4.5 ± 0.3	1.9 ± 0.3
FOS4	4.0 ± 0.3	2.1 ± 0.3
FOS7	3.9 ± 0.4	1.5 ± 0.5
FOS8	4.1 ± 0.1	2.0 ± 0.8
D457 Δ*zwf*	5.6 ± 0.8	2.6 ± 0.3
FOS4 Δ*zwf*	5.0 ± 0.5	4.9 ± 0.2
FOS7 Δ*zwf*	6.6 ± 0.7	4.7 ± 0.6

a Susceptibility to H_2_O_2_ and menadione was measured by the diameter (in centimeters) of the zone of growth inhibition around each disk.

### The impaired activity of EMP enzymes is associated with S. maltophilia fosfomycin resistance.

Our results strongly suggest that the cause of fosfomycin resistance in the mutants studied is a reduced activity of the enzymes of the lower glycolysis pathway in S. maltophilia. However, it is still possible that these enzymes may present moonlighting activities in this bacterial species besides its metabolic role, which could be associated with their AR phenotype in a metabolism-independent manner (46, 47). This possibility is supported by the fact that, while mutations in these genes were easily selected in S. maltophilia, the information present in the Profiling of the Escherichia coli Chromosome (PEC) database, the Keio library, and the Transposon-directed insertion site sequencing (TraDIS) database (48–50) supported that these genes are highly relevant (eventually essential) in E. coli.

**FIG 4:**
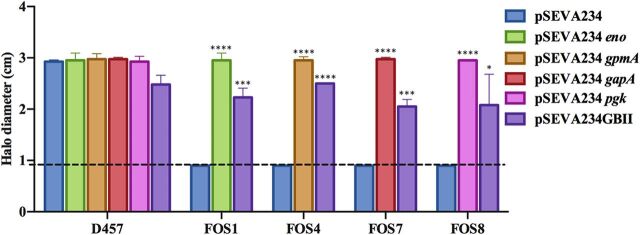
Fosfomycin susceptibility of the fosfomycin-resistant mutants complemented with either S. maltophilia D457 or E. coli K-12 enzymes. The halo inhibition diameter of fosfomycin disks is shown for the wild-type D457 strain and the four mutants. In all cases, the results are shown for the strains containing either the pSEVA234 backbone used for cloning, pSEVA234 containing the corresponding wild-type alleles of S. maltophilia genes (*eno*, *gpmA*, *gapA*, and *pgk*), or a partial version of Glucobrick II (GBII) with E. coli genes *gapA*, *pgk*, *gpmA*, and *eno.* The dashed line at 0.9 cm indicates the diameter of the disk. Error bars indicate standard deviations of the results from three independent replicates. Statistical significance calculated by unpaired two-tail *t* test: *, *P* < 0.02; ***, *P* < 0.0002; ****, *P* < 0.0001.

We then wanted to determine whether the recovery of glycolytic activity, independently of putative additional activity of the S. maltophilia inactivated enzymes, could be the basis of the observed AR phenotype. For this purpose, a partial version of the Glucobrick II, containing the Escherichia coli genes *gapA*, *pgk*, *gpmA*, and *eno* was introduced in the S. maltophilia fosfomycin-resistant mutants and in the wild-type strain, and the susceptibility to fosfomycin of these strains was measured. By this approach, the enzymatic activity, here provided by the E. coli orthologs of the S. maltophilia inactivated genes, was decoupled from another potential activity of such S. maltophilia proteins. As shown in Fig. 4, the expression of the E. coli GapA-Pgk-GpmA-Eno enzymes increased the susceptibility to fosfomycin of all FOS mutants, although the levels achieved were not the same as those of the wild-type strain. This partial complementation of the phenotype of resistance strongly supports that the absence of enzymatic activity of the analyzed EMP enzymes contributes to fosfomycin resistance in S. maltophilia.

### Fosfomycin resistance of mutants defective in EMP enzymes is not a consequence of increased production of PEP.

**FIG 5:**
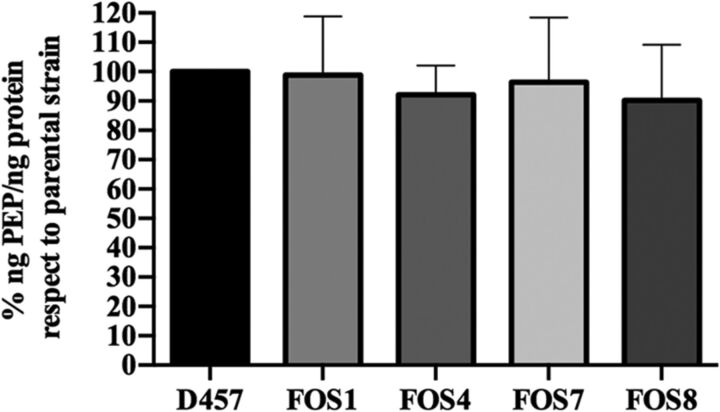
PEP intracellular concentration. The percentage of the concentration of intracellular PEP (in nanograms of PEP/nanograms of protein) in the fosfomycin-resistant mutants with respect to the value for the wild-type S. maltophilia D457 strain. Error bars indicate standard deviations for the results from three independent replicates.

Fosfomycin inhibits the action of MurA because it is structurally similar to PEP, one of the substrates of this enzyme. The EMP enzymes associated with fosfomycin resistance that are inactivated in the S. maltophilia fosfomycin-resistant mutants have reversible activity and belong to a pathway that leads to either PEP biosynthesis or consumption depending on the metabolic regime. It might be possible that inactivation of such enzymes may change the intracellular PEP concentrations, affecting the binding of fosfomycin to the active site of MurA through a possible competition between PEP and fosfomycin, which may result in reduced susceptibility to fosfomycin. To assess if this was the case, the concentration of PEP was analyzed in the wild-type D457 strain and in the fosfomycin-resistant mutants. As shown in Fig. 5, none of the mutants presented an increase in the intracellular concentration of PEP, ruling out the hypothesis that the reduced susceptibility to fosfomycin of the analyzed mutants was due to increased production of PEP.

### Fosfomycin resistance mutations impair the gluconeogenic pathway of S. maltophilia.

**FIG 6:**
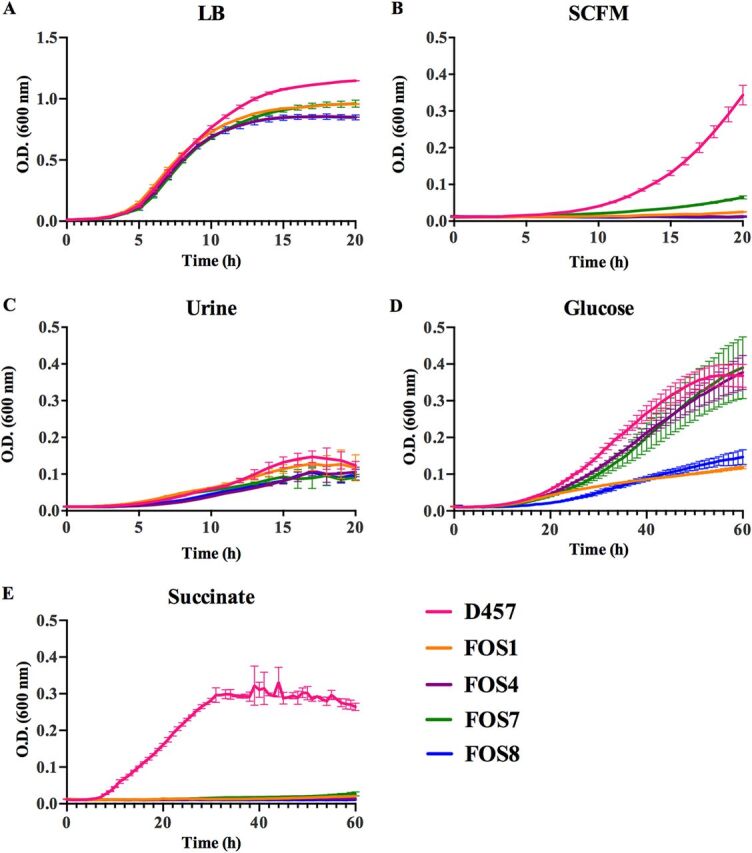
Effects of fosfomycin resistance mutations on the growth of S. maltophilia in either LB, clinical media, glucose, or succinate. Areas under the curves were calculated using GraphPad Prism. (A) Growth of the different strains in LB. Total area under the curve: 12.84 for D457, 11.53 for FOS1, 10.53 for FOS4, 10.99 for FOS7, and 10.45 for FOS8. (B) Growth of the different strains in SCFM. Total area under the curve: 1.71 for D457, 0.30 for FOS1, 0.25 for FOS4, 0.53 for FOS7, and 0.23 for FOS8. (C) Growth of the different strains in urine. Total area under the curve: 1.41 for D457, 1.34 for FOS1, 0.97 for FOS4, 1.08 for FOS7, and 0.99 for FOS8. (D) Growth in SMMM containing glucose (40 mM). Total area under the curve: 10.33 for D457, 3.75 for FOS1, 8.82 for FOS4, 8.62 for FOS7, and 3.69 for FOS8. (E) Growth in SMMM containing succinate (40 mM). Total area under the curve: 12.28 for D457, 0.82 for FOS1, 0.72 for FOS4, 0.95 for FOS7, and 0.64 FOS8. Statistical analysis was performed using unpaired two-tail *t* test. As shown, the fosfomycin-resistant mutants were strongly impaired for growing in succinate and SCFM (in all cases, *P* < 0.0001), whereas the impairment for growing in glucose, particularly of FOS4 and FOS7, as well as in LB, was lower although the differences were statistically significant (*P* < 0.0001). The areas under the curve of the mutants and the wild-type strain growing in urine were not statistically significant, indicating that the effects of these mutations on the growth of S. maltophilia in urine are limited. Error bars indicate standard deviations for the results from three independent replicates.

The mutations selected in the presence of fosfomycin compromise the activity of relevant enzymes of S. maltophilia central metabolism. It is then expected that this would have relevant physiological consequences. To determine the general scope of these consequences, the growth of S. maltophilia mutants and the wild-type parental strain was measured under different conditions. Only small differences in growth among the strains were observed for bacteria growing in rich LB medium (Fig. 6A), indicating that these mutations do not impose a relevant general, nonspecific, fitness cost. Nevertheless, these mutants present relevant impaired growth in synthetic sputum medium (SCFM), indicating that their growth can be compromised in the context of a respiratory infection (Fig. 6B). Opposite to this situation, differences in growth between the wild-type strain and the mutants were not observed when they grew in urine (Fig. 6C). These results suggest that the possibility of these mutants being maintained in the absence of selection will strongly depend on the type of S. maltophilia infection.

In addition, the mutants could grow using glucose as the sole carbon source, which imposes a glycolytic metabolism, although in the case of FOS1 and FOS8 at a different rate (Fig. 6D). Nevertheless, the mutants were unable to grow using succinate as the carbon source (Fig. 6E). This impaired growth in succinate was not observed when the mutants were complemented with either the wild-type allele of each of the mutated enzymes or the E. coli-derived Glucobrick II (Fig. S4). Blocking any of the enzymes of the EMP pathway between triose phosphate isomerase and pyruvate kinase breaks the amphibolic process in two branches. These branches work in opposite directions, starting either from glucose or from pyruvate to provide energy or biosynthetic intermediates (51). Since S. maltophilia also displays the one-direction ED pathway for glucose catabolism, mutants with low susceptibility to fosfomycin could grow, although at different rates, in minimal medium with glucose. Nevertheless, succinate as the sole carbon source did not support growth of the mutants because gluconeogenesis and, consequently, synthesis of hexose phosphates were impaired.

10.1128/mSystems.00282-20.4 FIG S4 The complementation with either the wild-type alleles of the mutated enzymes or the E. coli Glucobrick module II allows the recovery of the growth in succinate of the fosfomycin-resistant mutants. Growth in SMMM containing succinate (40 mM). (A) FOS1. Total area under the curve: 0.82 for FOS1 pSEVA234, 2.48 for FOS1 pSEVA234*eno*, and 8.91 for FOS1 pSEVA234GBII. (B) FOS4. Total area under the curve: 0.77 for FOS4 pSEVA234, 7.34 for FOS4 pSEVA234*gpmA*, and 8.52 for FOS4 pSEVA234GBII. (C) FOS7. Total area under the curve: 0.94 for FOS7 pSEVA234, 7.32 for FOS7 pSEVA234*gapA*, and 4.67 for FOS7 pSEVA234GBII. (D) FOS8. Total area under the curve: 0.82 for FOS8 pSEVA234, 7.00 for FOS8 pSEVA234*pgk*, and 8.97 for FOS8 pSEVA234GBII. In all cases, the differences in growth for each mutant and its complemented derivatives were statistically significant, with *P* < 0.0001 as determined using unpaired two-tail *t* test. (E) D457. Total area under the curve: 8.97 for D457 pSEVA234, 7.43 for D457 pSEVA234*eno*, 8.14 for pSEVA234*gpmA*, 7.28 for pSEVA234*gapA*, 7.12 for pSEVA234*pgk*, and 10.39 for pSEVA23GBII. Error bars indicate standard deviations for the results from three independent replicates. Download FIG S4, TIF file, 0.5 MB. Copyright © 2020 Gil-Gil et al. 2020 Gil-Gil et al. This content is distributed under the terms of the Creative Commons Attribution 4.0 International license.

### Fosfomycin-resistant mutants do not present an altered intracellular accumulation of fosfomycin.

We have determined that the primary cause of fosfomycin resistance in S. maltophilia is the inactivation of EMP enzymes. However, it may be possible that such inactivation impairs the accumulation of the antibiotic within the cell, which could be due to either reduced uptake or degradation of the antibiotic. A search of possible fosfomycin transporters in S. maltophilia D457 was conducted using BLAST (52) with the sequences of the fosfomycin transporters UhpT and GlpT. This search did not identify any possible transporter of hexose and triose phosphates in the genome of S. maltophilia D457, orthologs to those known in other microorganisms. Nevertheless, alternative transporters could still internalize these sugars. This possibility was tested by growing the different strains in S. maltophilia minimum medium (SMMM) containing either glucose-6P or glycerol-3P as the sole carbon source. Despite the fact that S. maltophilia harbors the orthologs of the enzymes required for the catabolism of glucose-6P and glycerol-3P, none of the strains was able to grow using these sugars as unique carbon sources, conditions under which E. coli can grow (Fig. S5). This result suggests that S. maltophilia lacks glucose-6P and glycerol-3P transporters, which are the regular gates for fosfomycin entry in other pathogens. Besides, orthologs of the genes encoding the fosfomycin resistance proteins (FosA, FosB, FosX, FomA, FomB, and FosC) so far described in the literature, were not detected in the genome of S. maltophilia D457.

10.1128/mSystems.00282-20.5 FIG S5 Growth of the fosfomycin-resistant mutants using phosphorylated glycolytic intermediates. (A) Growth in SMMM containing 40 mM glucose-6P for 60 h. Total area under the curve: 1.01 for D457, 0.87 for FOS1, 0.95 for FOS4, 0.99 for FOS7, 0.65 for FOS8, and 12.00 for E. coli K-12. (B) Growth in SMMM containing 40 mM glycerol-3P for 60 h. Total area under the curve: 1.89 for D457, 0.82 for FOS1, 0.76 for FOS4, 1.64 for FOS7, 1.21 for FOS8, and 14.15 for E. coli K-12. (C) Control of bacteria incubated in SMMM without a carbon source. Total area under the curve: 0.95 for D457, 0.82 for FOS1, 0.82 for FOS4, 0.65 for FOS7, 0.87 for FOS8, and 1.27 for E. coli K-12. As shown, S. maltophilia cannot grow using these carbon sources, whereas E. coli can grow using both carbon sources. Error bars indicate standard deviations for the results from three independent replicates. Download FIG S5, TIF file, 0.3 MB. Copyright © 2020 Gil-Gil et al. 2020 Gil-Gil et al. This content is distributed under the terms of the Creative Commons Attribution 4.0 International license.

**FIG 7:**
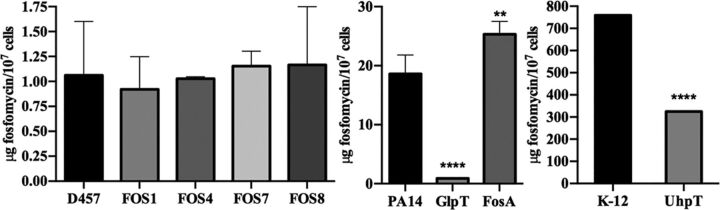
The intracellular concentration of fosfomycin does not change in the fosfomycin-resistant mutants. Comparison of the fosfomycin intracellular concentration between the mutants and the parental strain. There is not a deficiency in the fosfomycin transport in the mutants that determines its resistance or a fosfomycin-modifying enzyme involved in this resistance. PA14 and its mutants GlpT and FosA and E. coli K-12 and its mutant UhpT were used as controls of the assay. Error bars indicate standard deviations for the results from three independent replicates. Statistical significance calculated by unpaired two-tail *t* test: **, *P* < 0.001; ****, *P* < 0.0001.

Despite the fact that the S. maltophilia genome does not harbor genes encoding either the canonical fosfomycin transporters or already known fosfomycin-inactivating enzymes, it might be possible that other (still unknown) elements may contribute to impairing the accumulation of the antibiotic inside the mutants. To analyze this possibility, the intracellular accumulation of fosfomycin in the different strains was measured (53) after 1 h of incubation with 2 mg/ml fosfomycin in exponential-growth-phase cultures. For controls, E. coli K-12 and its deletion mutant with the fosfomycin transporter UhpT deleted (48), as well as P. aeruginosa PA14 and its mutants with insertions in the genes encoding the fosfomycin transporter GlpT and the fosfomycin resistance protein FosA (54) were used. As shown in Fig. 7, the amount of intracellular fosfomycin was lower in both E. coli and P. aeruginosa when their respective fosfomycin transporters (GlpT and UhpT) are inactivated. Conversely, an increased fosfomycin concentration was observed in the FosA mutant relative to the parental PA14 strain, which supports the validity of these assays. Nevertheless, the intracellular concentrations of fosfomycin were similar in the wild-type S. maltophilia D457 strain and in the isogenic fosfomycin-resistant mutants. These results suggest that the resistance to fosfomycin of the FOS mutants is not due to reduced intracellular concentration of this antibiotic. Notably, fosfomycin accumulation in S. maltophilia was much lower than that found in E. coli or P. aeruginosa. Indeed, intracellular fosfomycin concentration in S. maltophilia was in the range observed for the GlpT-defective P. aeruginosa mutant. This low intracellular concentration, likely associated with the lack of canonical antibiotic transporters, could be the cause of the intrinsic lower susceptibility of S. maltophilia D457 to fosfomycin compared to E. coli K-12 and P. aeruginosa PA14 (53, 55, 56).

### Effects of fosfomycin resistance mutations on the transcriptional profile of S. maltophilia.

**FIG 8:**
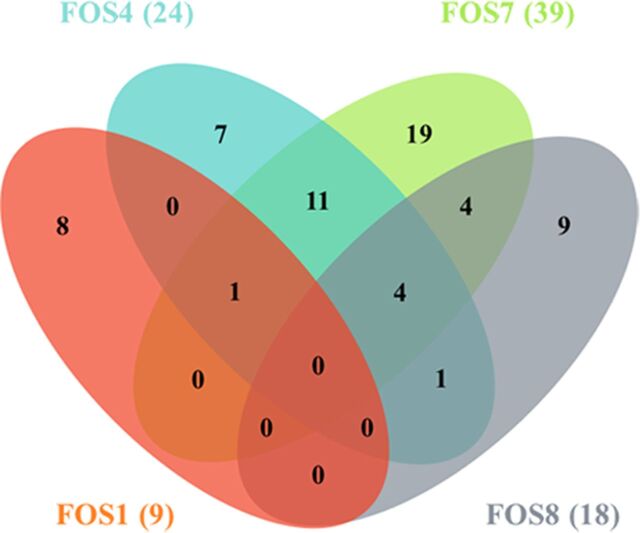
Common and differential transcriptomic changes of the fosfomycin-resistant mutants. The figure presents a Venn diagram showing the number of genes whose expression changes in the mutants relative to the parental strain were relevant (≥1 log_2_ fold or ≤−1 log_2_ fold). The number of genes with changes in each mutant is shown in parentheses. As shown, most transcriptomic changes are specific for each analyzed mutant.

In order to know whether the mutation of genes encoding the enzymes involved in central carbon metabolism changes the transcriptional profile in a way directly related to fosfomycin resistance, the transcriptomes of the fosfomycin-resistant mutants were compared to that of the wild-type strain. Changes in the expression ratios (≥2-fold or ≤0.5-fold) of just 64 of the 4,210 genes that form the genome of S. maltophilia D457 were detected (see Table S1 in the supplemental material). Most changes were specific for each mutant, indicating that the observed transcriptomic changes were unlikely associated with the common phenotype of fosfomycin resistance (Fig. 8). Concerning changes that may explain the resistance phenotype, it is important to note the absence of relevant transcriptional changes in genes related to cell wall synthesis, such as the gene encoding the fosfomycin target MurA and SMD_1053, SMD_1054, SMD_0334, *nagZ*, and SMD_2885, predicted to be involved in recycling peptidoglycan (Table 6). These results support that increased expression of either the fosfomycin target (MurA) or the alternative peptidoglycan recycling pathway is not the cause of fosfomycin resistance in the mutants analyzed.

**TABLE 6:** Fold change of *murA* and genes involved in recycling of the peptidoglycan in mutant strains compared to the wild-type D457 strain by RNA-Seq

Mutant strain	Fold change (log_2_) of the following gene:					
	*murA*	SMD_1053	SMD_1054	SMD_0334	*nagZ*	SMD_2885
FOS1	−0.13	−0.28	−0.23	−0.60	−0.27	−0.36
FOS4	0.01	−0.04	−0.18	−0.45	0.05	0.10
FOS7	−0.06	−0.10	−0.29	0.07	0.05	0.27
FOS8	−0.05	−0.26	−0.44	0.23	0.08	0.13

10.1128/mSystems.00282-20.6 TABLE S1 RPKM and fold change (log_2_) of genes presenting different levels of expression in the mutant strains compared to the wild-type D457 strain. Download Table S1, DOCX file, 0.04 MB. Copyright © 2020 Gil-Gil et al. 2020 Gil-Gil et al. This content is distributed under the terms of the Creative Commons Attribution 4.0 International license.

## DISCUSSION

Fosfomycin resistance mechanisms described so far have been clustered into three classical categories of AR acquisition (1): alterations in fosfomycin transport, antibiotic inactivation, and alterations in the target enzyme or peptidoglycan biosynthesis (17). Herein, using a set of *in vitro*-selected mutants, we have shown that none of these already known mechanisms seem to be involved in the acquisition of mutation-driven fosfomycin resistance by S. maltophilia. In this microorganism, the acquisition of resistance is due to the inactivation of enzymes belonging to the EMP pathway.

Our results indicate that the inactivation of these enzymes does not cause major changes in the transcriptomes of the mutants that may justify resistance as the consequence of a collateral effect of the selected mutations on the expression of the aforementioned fosfomycin resistance mechanisms. Intracellular accumulation of fosfomycin was similar in the wild-type and mutant strains, which support that resistance is neither due to an impaired fosfomycin uptake (25) nor to its degradation via the activity of fosfomycin-inactivating enzymes (29, 57). Also supporting this result is the fact that the genome of S. maltophilia does not encode orthologs of the already known fosfomycin resistance proteins or its transporters GlpT and UhpT.

Other mechanisms leading to fosfomycin resistance are modifications of the target MurA (21) or changes in its expression level. Nevertheless, when the mutants were sequenced, no mutations in *murA* were found, and analysis of the transcriptomes indicates that *murA* is not expressed at higher levels in the resistant mutants than in the wild-type strain. The same is true for the pathway involved in recycling peptidoglycan, where increased expression may contribute to fosfomycin resistance (7). Expression of the genes encoding the enzymes of this pathway is not higher in the mutants than in the wild-type strains as shown in the transcriptomic studies.

Therefore, classical AR mechanisms (1, 58) do not seem to be the cause of fosfomycin resistance in S. maltophilia. Although, at stated above, there are not relevant transcriptional changes in the mutants, these strains appear to be in a different physiological state than the wild-type strain, as evidenced by the fact that they exhibit increased Zwf activity together with the loss of function of the mutated enzymes. These changes do not modify the response to oxidative stress, an element that could be relevant in the activity of antibiotics (43). However, it is worth mentioning that regulation of the metabolic fluxes of carbon metabolism includes additional mechanisms other than transcriptional regulation (59). Among these mechanisms, allosteric regulation as well as the activity of posttranscriptional or posttranslational regulators can change the production levels and activity of different proteins (eventually involved in the resistance phenotype) without changing their mRNA levels (60).

The mutated enzymes belong to the amphibolic metabolic pathway (EMP and gluconeogenesis), which includes PEP, the natural substrate of MurA. Fosfomycin, due to its structural similarities to PEP, binds and inhibits MurA. It might then be possible that inactivation of such enzymes in the fosfomycin-resistant mutants may produce increased synthesis of PEP that could outcompete fosfomycin for its binding to MurA. However, the concentration of PEP is not higher in the fosfomycin-resistant mutants than in the wild-type strain, evidence against this possibility. Several enzymes from central metabolism are moonlighting proteins; they display functions unrelated to their enzymatic activity (46). The complementation of the mutants with E. coli enzymes restored their susceptibility to fosfomycin, which indicates that the impaired activity of these metabolic enzymes is the basis of the observed phenotype of fosfomycin resistance. Nevertheless, an alternative activity of the S. maltophilia enzymes unrelated to their known metabolic function cannot be totally discarded.

Previous analysis has shown that E. coli mutants deficient in the metabolic enzyme isocitrate dehydrogenase are resistant to nalidixic acid (35). However, there has been little work on the cross talk between metabolism (and metabolic robustness) and AR (61, 62), despite the fact that metabolic interventions may improve the activity of the antibiotics (33, 63–65) and that bacterial metabolism can constrain the evolution of AR (13). Our results highlight the importance that the modification of the activity of enzymes belonging to central metabolism may have on the susceptibility to antibiotics, such as fosfomycin, that are not known to interact with such enzymes. The finding that fosfomycin activity is highly dependent on the bacterial metabolic status, being more active when bacteria grow intracellularly (27, 28) or under acidic conditions and anaerobiosis in urine (66), further support that antibiotic activity and, consequently AR, are interlinked with bacterial metabolism.

## MATERIALS AND METHODS

### Bacterial strains and culture conditions.

All bacterial strains, plasmids, and oligonucleotides used in this study are listed in Tables S2 and S3 in the supplemental material. Unless otherwise stated, bacteria were grown in LB (lysogeny broth) Lennox medium at 37°C with constant agitation at 250 rpm. Solid medium was prepared using an agar concentration of 15 g/liter. In order to analyze the growth of S. maltophilia D457 in the presence of a single carbon source, S. maltophilia minimum medium (SMMM) (67) with modifications was used. SMMM contained 500 mg/liter K_2_HPO_4_, 500 mg/liter KH_2_PO_4_, 800 mg/liter NH_4_HPO_4_, 200 mg/liter MgSO_4_, 53 mg/liter CaCl_2_, 0.85 mg/liter MnSO_4_, 0.5 mM l-methionine, 6.25 × 10^−3 ^ml/liter stock salts (10.75 mg/liter MgO, 2 mg/liter CaCO_3_, 4.5 mg/liter FeSO_4_·7H_2_O, 1.44 mg/liter ZnSO_4_·7H_2_O, 1.11 mg/liter MnSO_4_·4H_2_O, 0.25 mg/liter CuSO_4_·5H_2_O, 0.28 mg/liter CoSO_4_·7H_2_O, 0.06 mg/liter H_3_BO_3_·7H_2_O, 51.3 ml/liter HCl), 10 μM ammonium ferric citrate, and 0.01% Casamino Acids. The carbon source was added in each case at 40 mM. The effects of the mutations on fitness in infection contexts was also tested by growing the different strains in either urine or synthetic cystic fibrosis sputum medium (SCFM). Urine was obtained from five healthy volunteers, mixed, and filtered to remove any potential bacterial contamination. SCFM was prepared as described by K. L. Palmer et al. (68). When required, antibiotics were added: 100 μg/ml ampicillin and 50 μg/ml kanamycin for E. coli, and 500 μg/ml kanamycin for S. maltophilia. Different concentrations of fosfomycin as well as 1 mM IPTG were used in different experiments, as stated in the different sections.

10.1128/mSystems.00282-20.7 TABLE S2 Bacterial strains and plasmids used in this work. Download Table S2, DOCX file, 0.04 MB. Copyright © 2020 Gil-Gil et al. 2020 Gil-Gil et al. This content is distributed under the terms of the Creative Commons Attribution 4.0 International license.

10.1128/mSystems.00282-20.8 TABLE S3 Oligonucleotides used in this work. Download Table S3, DOCX file, 0.02 MB. Copyright © 2020 Gil-Gil et al. 2020 Gil-Gil et al. This content is distributed under the terms of the Creative Commons Attribution 4.0 International license.

### Isolation of fosfomycin-resistant mutants.

Around 10^8^ S. maltophilia D457 bacterial cells were plated on Mueller-Hinton agar petri dishes containing 1,024 μg/ml fosfomycin and were grown at 37°C during 48 h. The mutants selected under these conditions were grown on LB agar without antibiotic (three sequential passages) and then were grown on LB agar containing 1,024 μg/ml fosfomycin to ensure that the observed phenotype was not transient. The susceptibility of mutants to fosfomycin was tested (see below), for further studies 4 mutants were randomly selected and dubbed FOS1, FOS4, FOS7 and FOS8.

### DNA extraction, whole-genome sequencing, and SNP identification.

Chromosomal DNA from each mutant (FOS1, FOS4, FOS7, and FOS8) and the wild-type strain (D457) was obtained from overnight cultures using the Gnome DNA kit (MP Biomedicals). DNA libraries were prepared using the TruSeq DNA sample preparation v.2 kit and were sequenced in a MiSeq Illumina instrument at the Parque Científico de Madrid, Spain. The samples were subjected to single-end sequencing with a read length of 150 bp, and a coverage between 26 and 41× was obtained. The genomic sequences of the strains were compared to the S. maltophilia D457 reference genome (NC_017671.1) and visualized using the software FIESTA 1.1 (http://bioinfogp.cnb.csic.es/tools/FIESTA). Mutations were filtered according to sequence quality (>30) and the mutation effect in the protein sequence (moderate and high effect), and the variants absent in the control D457 parental strain were studied. Provean predictor (provean.jcvi.org) was used to anticipate whether an amino acid substitution or indel had an impact on the biological function of the coding protein.

The mutations detected by the whole-genome sequencing analysis were confirmed by PCR and Sanger sequencing. For this purpose, the glycolytic genes (*eno* for FOS1, *gpmA* for FOS4, *gapA* for FOS7, and *pgk* for FOS8) were amplified by PCR using the primers indicated in Table S3. PCR products from DNA from mutant strains were purified with the purification kit from GE Healthcare and Sanger sequenced by StabVida (Caparica, Portugal).

### Antimicrobial susceptibility assays.

The gentamicin, tobramycin, ciprofloxacin, nalidixic acid, ceftazidime, colistin, tetracycline, chloramphenicol, and fosfomycin MICs were determined for each strain on LB agar using MIC test strips (MIC Test Strips; Liofilchem Diagnostics). For the phenotypic analysis of the mutants complemented with the Glucobrick module II, paper disks (9 mm; Macherey-Nagel) impregnated with 0.5 mg of fosfomycin were used. Plates were incubated at 37°C, and results were analyzed after 20 h. The experiments were performed in triplicate.

### Complementation of fosfomycin-resistant mutants and generation of *zwf* deletion mutants.

The genes *eno*, *gpmA*, *pgk*, and *gapA*, encoding glycolytic enzymes, were obtained from the wild-type S. maltophilia D457 strain by PCR amplification using the primers shown in Table S3. Amplicons of each complete gene (*eno*, *gpmA*, *pgk*, and *gapA*) obtained from wild-type S. maltophilia D457 were cloned in the pGEM-T Easy vector (Promega) following the protocol provided by the manufacturer. The ligation products were introduced, by transformation, into One Shot OmniMAX Chemically Competent E. coli (Invitrogen). The transformed cells were poured in LB plates containing ampicillin, and colonies that grew after 24 h were selected and used in colony PCR amplifications (69) with M13 primers (Table S3) to check for the presence of plasmids deriver from pGEM-T Easy. The inserted fragments were Sanger sequenced by StabVida (Caparica, Portugal) to ensure that no mutations were introduced during PCR. Plasmids were purified with the QIAprep Spin Miniprep kit and digested with New England Biolabs restriction enzymes EcoRI and HindIII. The DNA fragments corresponding to the inserts (*eno*, *gpmA*, *pgk*, or *gapA*) were purified with the purification kit from GE Healthcare. Then, ligation reactions were done with the pSEVA234 plasmid digested with the same enzymes and the purified inserts using T4 DNA ligase (BioLabs Inc.) at 16°C overnight. The resulting plasmids (pSEVA234*eno*, pSEVA234*gpmA*, pSEVA234*pgk*, and pSEVA234*gapA*) as well as the cloning vector pSEVA234 were introduced into E. coli CC118λpir competent cells and then into S. maltophilia D457 and the FOS1, FOS4, FOS7, and FOS8 mutants by triple conjugation (70). Exconjugants were selected in LB plates containing 500 μg/ml kanamycin and 20 μg/ml imipenem. The presence of the pSEVA234 plasmid and the insert in the plasmid in isolated exconjugant colonies was confirmed by PCR with primers 227 and 273 and pSEVA234_F and pSEVA234_R, respectively (Table S3).

To complement the mutants with a partial version of Glucobrick module II, which contains the genes encoding the enzymes of the lower glycolytic pathway of E. coli K-12 (*gapA*, *pgk*, *gpmA*, *eno*, and *pyk*) (71), the pSEVA224-GBII plasmid containing these genes was purified with the QIAprep Spin Miniprep kit and digested with restriction enzymes BamHI and HindIII, obtaining the *gapA-pgk-gpmA-eno* fragment of Glucobrick module II. The corresponding band was purified and ligated into pSEVA234 previously digested with the same enzymes. The new pSEVA234(*gapA-pgk-gpmA-eno*) plasmid was introduced into S. maltophilia strains D457, FOS1, FOS4, FOS7, and FOS8 by triple conjugation (70).

The *zwf* gene was deleted in different S. maltophilia strains by homologous recombination as described previously (72). To delete the *zwf* gene, a 500-bp fragment (ZwfA) corresponding to the 5′ end of *zwf* was amplified using primers ZwfAF and ZwfAR (Table S3). Another 500-bp fragment (ZwfB) of the *zwf* 3′ end was amplified using primers ZwfBF and ZwfBR (Table S3). Using the ZwfA and ZwfB amplicons as the templates, an overlapping PCR was conducted using primers ZwfAF and ZwfBR, yielding a 1,000-bp fragment (ZwfAB). The product obtained was cloned into pGEM-T Easy (Promega) and introduced by transformation into E. coli CC118λ*pir*. DNA sequencing was performed for sequence verification. The plasmid was then digested with EcoRI, and the ZwfAB fragment was cloned into pEx18Tc. The resulting plasmid pTGG05 was introduced by transformation into E. coli CC118*λpir* and then, by tripartite matting (70), into S. maltophilia D457, FOS4, and FOS7. The *zwf*-defective mutants were selected as described previously (72), and confirmation of the deletion in S. maltophilia FOS4 and FOS7 strains was performed using the primers IntZwf_F and IntZwf_R, as well as ExtZwf_F and ExtZwf_R (Table S3).

### RNA extraction and RNA-Seq.

The different bacterial strains were grown overnight in LB broth at 37°C and 250 rpm. These cultures were used to inoculate new flasks to reach an optical density at 600 nm (OD_600_) of 0.01, and the cultures were grown at 37°C until an OD_600_ of 0.6 was reached. Afterwards, RNA was isolated (73). Twenty milliliters of each bacterial culture was spun down at 6,000 × *g* for 3 min at 4°C and immediately frozen on dry ice and stored at −80°C. RNA was isolated from cell pellets using the RNeasy minikit (Qiagen) following the manufacturer’s instructions. To remove the remaining genomic DNA, total RNA samples were treated with DNase I (RNase-Free DNase Set [Qiagen]), and a second digestion was then performed following the Turbo DNA-free (Ambion) protocol. The RNA was purified and concentrated using RNeasy minikit columns. Finally, DNA contamination was checked by PCR with primers 27 and 48 (Table S3). Only RNAs containing no DNA contamination were used for further studies.

To analyze the transcriptome of S. maltophilia D457 and mutant strains, RNA was obtained from three independent cultures of each strain, which were then pooled to reduce biological variability. Libraries were prepared using 5 μg of RNA, rRNA was depleted using RiboZero kit, and cDNA was synthesized. The libraries were prepared using TruSeq v.2 kit. Transcriptome sequencing (RNA-Seq) was conducted at Sistemas Genómicos S.L., Parque Tecnológico de Valencia, with Illumina HiSeq 2500 sequencing technology using a 50-bp single-end format. Reads per kilobase per million mapped read (RPKM) values were obtained using Rockhopper (74). Fold change was calculated as the logarithm in base 2 of the quotient between the RPKM value of the mutant strain and the RPKM value of the wild-type strain for each gene. A minimum of 15 RPKM in at least one of the strains analyzed was used as the threshold to include the corresponding genes in the analysis. Only fold changes of <−1 or >1 were considered relevant values. A Boolean analysis of RPKM fold change relevant values for each gene, of each mutant FOS1, FOS4, FOS7, and FOS8 relative to the parental strain was performed using the Venny tool (75).

### Bacterial growth measurements.

Growth was measured with a Spark 10M plate reader (Tecan) at OD_600_ in flat-bottomed transparent 96-well plates (Nunc MicroWell; Thermo Fisher). Each well was inoculated with bacterial suspensions to a final OD_600_ of 0.01 in LB, urine, SCFM, or SMMM containing a 40 mM concentration of the carbon source under study. For SMMM experiments, overnight cultures were washed twice with SMMM medium without any carbon source. The plates were incubated at 37°C with 10 s of shaking every 10 min. In all cases, a noninoculated well containing the corresponding medium was included as a test of medium sterility.

### Protein quantification.

Protein concentration was determined following the Pierce BCA Protein assay kit (Thermo Scientific) protocol in 96-well plates (Nunc MicroWell; Thermo Fisher).

### *In vitro* activity assays of the enzymes of the lower glycolytic pathway and dehydrogenases.

Cells were harvested at exponential phase (OD_600_ = 0.6) by centrifugation at 5,100 × *g* and 4°C and washed twice in 0.9% NaCl and 10 mM MgSO_4_. Once washed, cells were disrupted by sonication at 4°C, and the cell extracts were obtained by centrifugation at 23,100 × *g* for 30 min at 4°C.

NAD(P)^+^ reduction or NAD(P)H oxidation was monitored spectrophotometrically at 340 nm and 25°C with intermittent shaking in microtiter plates using a Spark 10M plate reader (Tecan). Each reaction was performed using three biological replicates, and the specific activities were obtained by dividing the measured slope of NAD(P)H formation or consumption by the total protein concentration. Enzymatic activities of dehydrogenases (glucose-6-phosphate, isocitrate and glyceraldehyde-3P dehydrogenases) were measured as described previously (76).

Enzymatic activities of phosphoglycerate kinase, phosphoglycerate mutase, and enolase were assayed following the protocol described by A. Pawluk et al. (77) with some modifications in a two-step reaction. In all cases, the first step in the determination of enzymatic activity was performed by adding 10 μl of the cell extract to 90 μl of K/Mes buffer (30 mM KCl and 3 mM MgCl_2_), pH 6.5, and the corresponding substrates for each enzyme (see below). After 15 min of incubation at room temperature, the mixtures were heated for 1 min at 95°C to stop the reaction, and the second step was performed. In all cases, NAD(P)^+^ reduction or NAD(P)H oxidation was monitored spectrophotometrically. Phosphoglycerate kinase was assayed in a first step containing 5 mM 3-phosphoglycerate, 1 mM ATP, and 10 mM β-mercaptoethanol, and in a second step, the formation of glyceraldehyde-3P was measured by adding 0.15 mM NADH and 10 U/ml of glyceraldehyde-3P dehydrogenase. For the phosphoglycerate mutase, the reaction mixture of the first step contained 0.5 mM ADP and 10 mM β-mercaptoethanol, and in the second step, the formation of lactate was measured by adding 0.15 mM NADH and 10 U/ml each of enolase, pyruvate kinase, and lactate dehydrogenase. For determining enolase activity, the mixture of the first step contained 30 mM triethanolamine, 0.4 mM 2-phosphoglycerate, and 0.5 mM ADP, and in the second step, the formation of lactate was measured by adding 0.15 mM NADH and 10 U/ml each of pyruvate kinase and lactate dehydrogenase.

### H_2_O_2_ and menadione susceptibility test.

The susceptibility to H_2_O_2_ and menadione was tested as described previously with some modifications (78). Sterile paper disks (9 mm; Macherey-Nagel) were impregnated with 10 μl of 2.5% H_2_O_2_ or 20 μl of 0.2 M menadione and placed on LB agar plates. The diameter of the zone of growth inhibition around each disk was measured after 20 h of incubation at 37°C. The experiment was performed in triplicate.

### Quantification of intracellular phosphoenolpyruvate.

The amount of PEP was measured from cultures in exponential growth phase in LB medium (OD_600_ = 0.6). Twenty milliliters of each culture was centrifuged at 4,500 × *g* for 3 min at 4°C. PEP Colorimetric/Fluorometric assay kit protocol (Sigma-Aldrich) was used with some modifications. For measuring PEP concentrations, bacterial pellets from exponential cultures were frozen with liquid nitrogen. Once the pellets were frozen, 200 μl of 3 M perchloric acid was added at 4°C, and the cells were disrupted by vortexing. The mixture was then neutralized by the addition of 1 M potassium bicarbonate until the pH reached 6.5 to 7.5, making use of the vortex between each addition of 50 μl of the potassium bicarbonate solution. The mixtures were centrifuged at 15,700 × *g* for 5 min at 4°C. The supernatants were transferred to new microcentrifuge tubes and used immediately.

For the measurement of PEP, 96-well plates with transparent bottom and dark walls (Costar assay plate) were used. A total of 25 μl of the reaction mixture (22 μl PEP buffer, 1 μl PEP probe, 1 μl PEP converter, and 1 μl PEP developer) and 25 μl of the corresponding supernatant were inoculated into each well. The concentration of PEP was determined by an enzyme-coupled assay after 60 min of incubation at room temperature, in which the PEP is converted to ATP and pyruvate, resulting in a fluorometric product (excitation wavelength [λ_exc_] of 535 and emission wavelength [λ_em_] of 587 nm) proportional to the amount of PEP present in the sample. The fluorescence was measured using optimal gain in a Spark 10M plate reader (Tecan). The concentration of PEP in the sample was calculated with a calibration line and normalized with the amount of protein in each of the samples.

### Quantification of intracellular fosfomycin.

Assays to test fosfomycin accumulation in bacterial cells were conducted as previously stated (53), with some modifications. For measuring the amount of intracellular concentration of fosfomycin, bacteria were grown in 40 ml of LB medium to exponential phase, and after centrifugation (4,500 × *g* 3 min), the bacteria were resuspended in 2 ml of LB medium. These suspensions were incubated for 60 min at 37°C in the presence of 2 mg/ml of fosfomycin and then washed three times with 1 ml of buffer (10 mM Tris [pH 7.3], 0.5 mM MgCl_2_, and 150 mM NaCl) to remove the extracellular fosfomycin. This fosfomycin concentration does not affect S. maltophilia mortality significantly. The cells were resuspended in 0.6 ml of 0.85% NaCl, and sequential dilutions of the suspensions were plated onto LB agar to determine the number of CFU per milliliter. The bacterial resuspension was boiled at 100°C for 5 min to release the intracellular fosfomycin. Ten microliters of each boiled suspension was plated onto LB agar as a death control. No colonies were obtained after 20 h at 37°C in any of the samples. In addition, as a degradation control, 40 μl of fosfomycin (50 mg/ml) was boiled at 100°C for 5 min. The activity of boiled fosfomycin did not change when determined using the bioassay described below.

After centrifugation (11,900 × *g*, 10 min), the antibiotic concentration in the supernatant was determined by a disk diffusion assay for each of the boiled suspensions. In this assay, sterilized assay disks (9 mm; Macherey-Nagel) were impregnated with 40 μl of either the supernatant or the control boiled fosfomycin and deposited onto LB agar plates overlaid with a 1:1,000 dilution of a culture of OmniMAX E. coli as a reporter strain grown overnight. The plates were incubated for 20 h at 37°C. The halo inhibition diameters of disks impregnated with different amounts of commercial fosfomycin, from 0.625 to 10 μg, were used to trace a standard curve in order to calculate the fosfomycin concentration in each sample.

The fosfomycin concentration in the supernatants was quantified by measuring the diameter (in centimeters) of inhibitory rings on the LB agar plates and was represented as micrograms per 10^7^ cells.

### Clinical isolate gene alignment.

Thirty-nine genomes of S. maltophilia isolates available in NCBI were selected for their clinical origin. *eno*, *gpmA*, *gapA*, and *pgk* sequences of all the selected isolates were aligned and compared to that of the wild-type D457 strain using Clustal Omega (79). Gene sequences were translated to protein using ExPASy bioinformatic resources portal (80). Protein sequences were aligned and compared to that of the wild-type D457 strain and FOS mutants using Clustal Omega.

### Metabolic map of S. maltophilia.

To model the metabolic map of S. maltophilia D457, indicating possible enzymes of the central metabolism and route bypasses, the BioCyc database (81) was used. The sequences of the enzymes were obtained from the complete genome of S. maltophilia D457 (82). In addition, the amino acid sequences of the enzymes of central metabolism of P. aeruginosa PAO1 (83) and E. coli (84) were aligned using the BLAST tool (52) with the S. maltophilia D457 genome confirming the presence or absence of these enzymes.

Moreover, a BLAST search was used to identify possible peptidoglycan recycling pathway genes, fosfomycin transporters, and fosfomycin resistance proteins in S. maltophilia D457.

### Data availability.

DNA sequences reported in this paper have been deposited in the Sequence Read Archive (SRA) database under BioProject accession number PRJNA628945, with the indicated SRA accession numbers for the following mutants: FOS1, SRX8197527; FOS4, SRX8197528; FOS7, SRX8197529; FOS8, SRX8197530. RNA-Seq data are available in Gene Expression Omnibus platform under accession number GSE141276. The GEO accession numbers for each sample follow: wild-type strain, GSM4200325; FOS1, GSM4200326; FOS4, GSM4200327; FOS7, GSM4200328; FOS8, GSM4200329.
