# Supplementary material for: The Inactivation of Enzymes Belonging to the Central Carbon Metabolism Is a Novel Mechanism of Developing Antibiotic Resistance
Source: mSystems. 2020 Jun 2;5(3):e00282-20. doi: 10.1128/mSystems.00282-20 (PMC8534728; doi:10.1128/mSystems.00282-20)
Supplement: FIG S2 [file msystems.00282-20-sf002.pdf]

A

Enolase

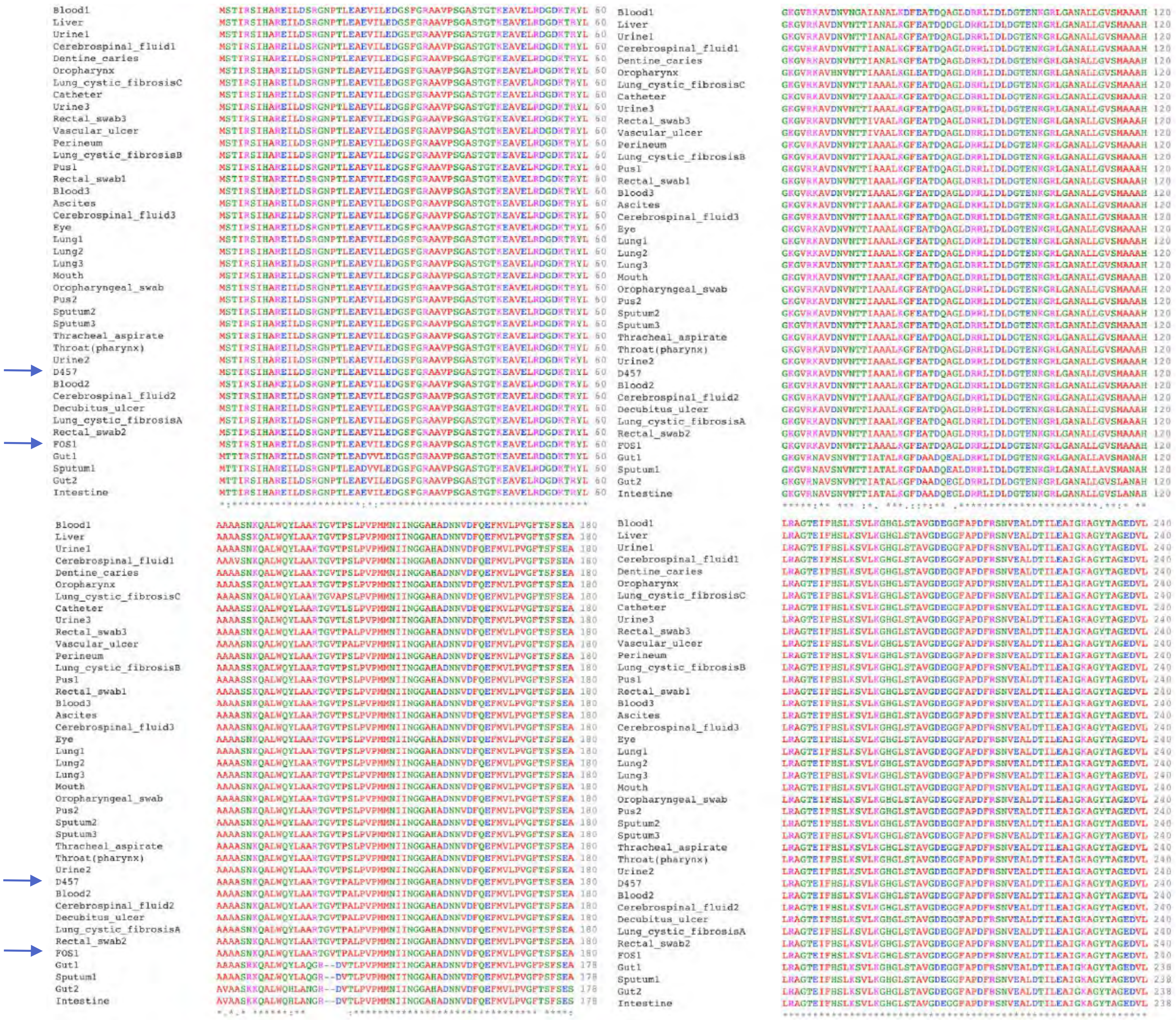

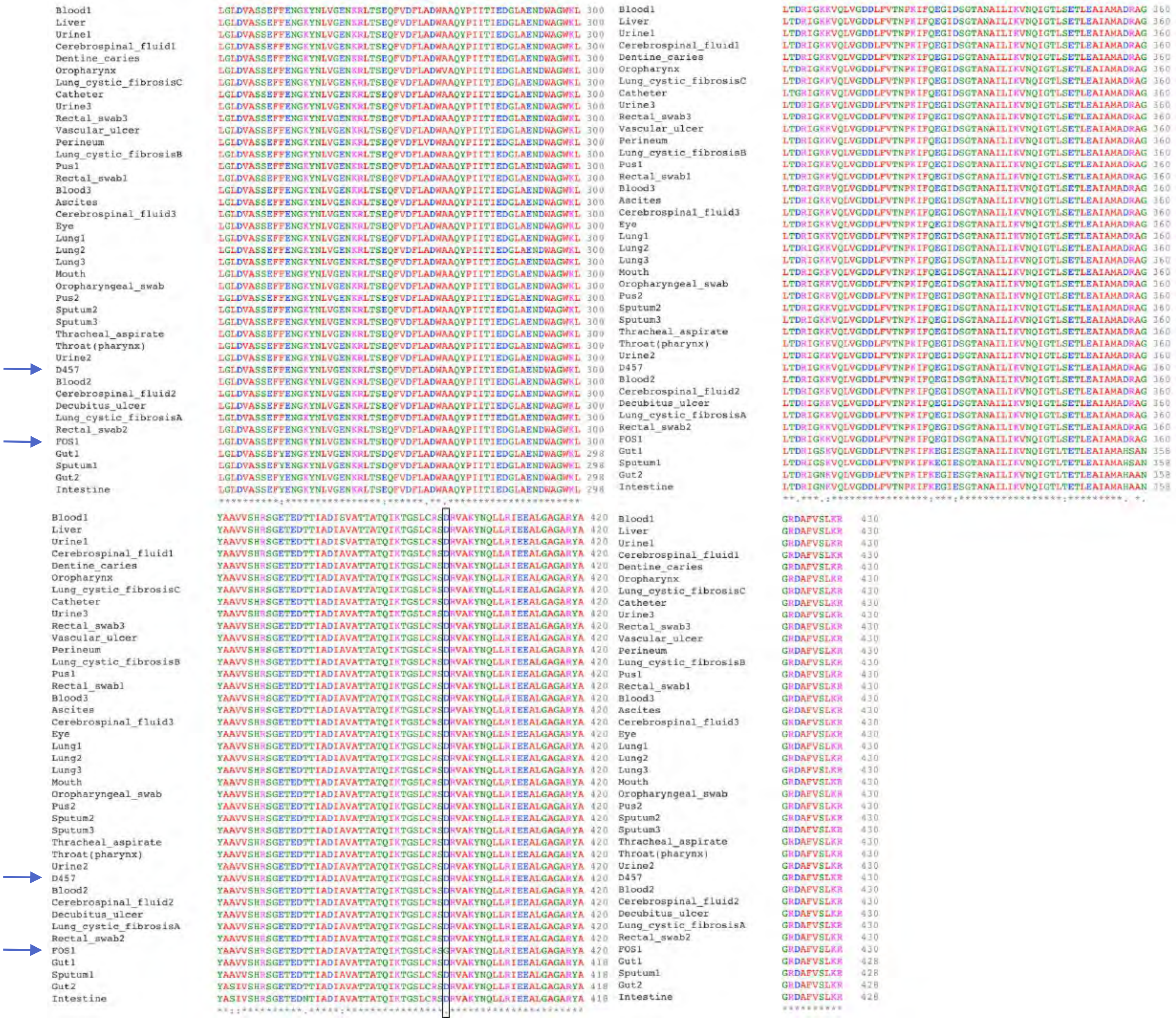
Blood1
Liver
Urine1
Cerebrospinal_fluid1
Dentine_caries
Oropharynx
Lung_cystic_fibrosisC
Catheter
Urine3
Rectal_swab3
Vascular_ulcer
Perineum
Lung_cystic_fibrosisB
Pus1
Rectal_swab1
Blood3
Ascites
Cerebrospinal_fluid3
Eye
Lung1
Lung2
Lung3
Mouth
Oropharyngeal_swab
Pus2
Sputum2
Sputum3
Tracheal_aspirate
Throat(pharynx)
Urine2
D457
Blood2
Cerebrospinal_fluid2
Decubitus_ulcer
Lung_cystic_fibrosisA
Rectal_swab2
FOS1
Gut1
Sputum1
Gut2
Intestine

B

Phosphoglycerate mutase

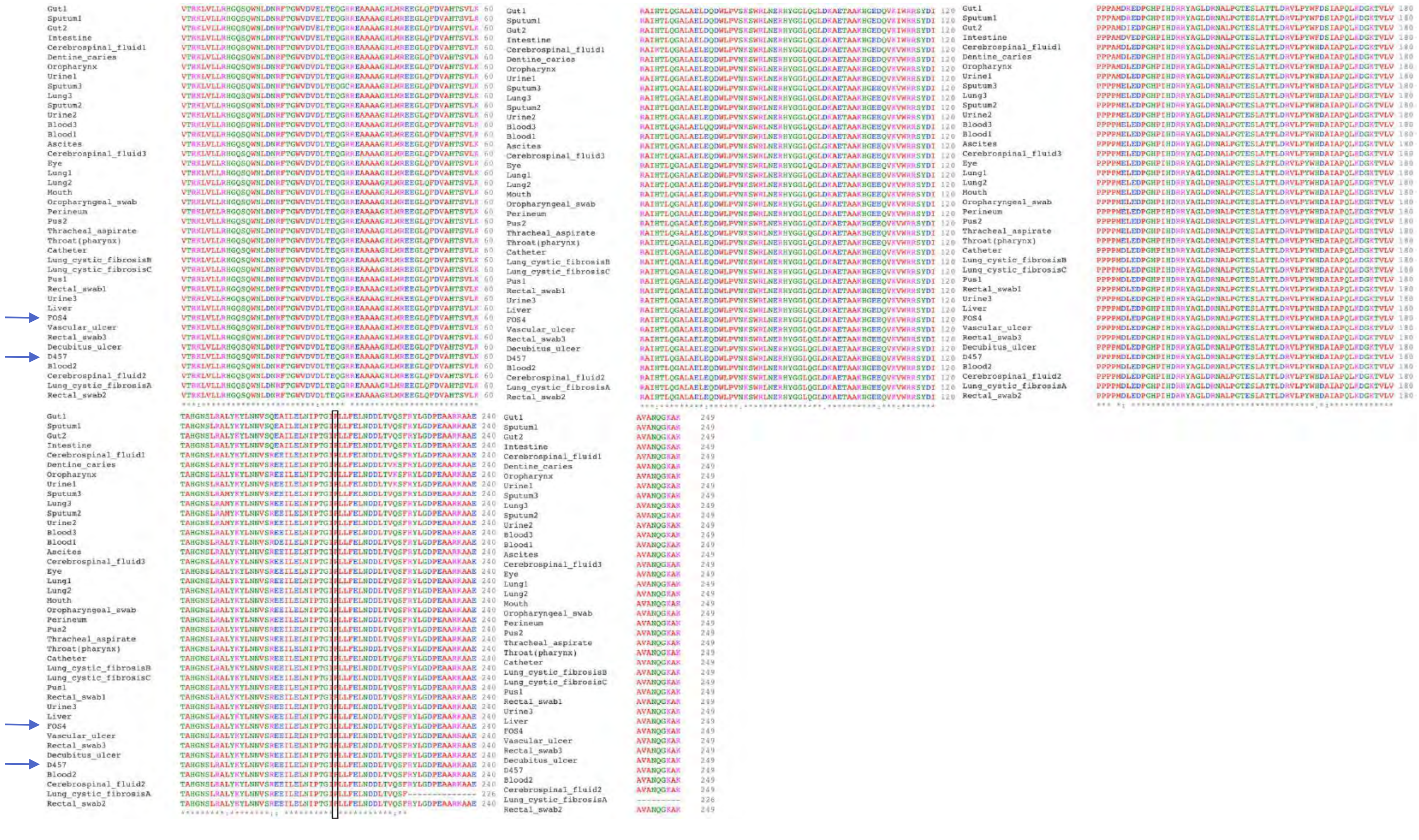

C **Glyceraldehyde-3-phosphate dehydrogenase**

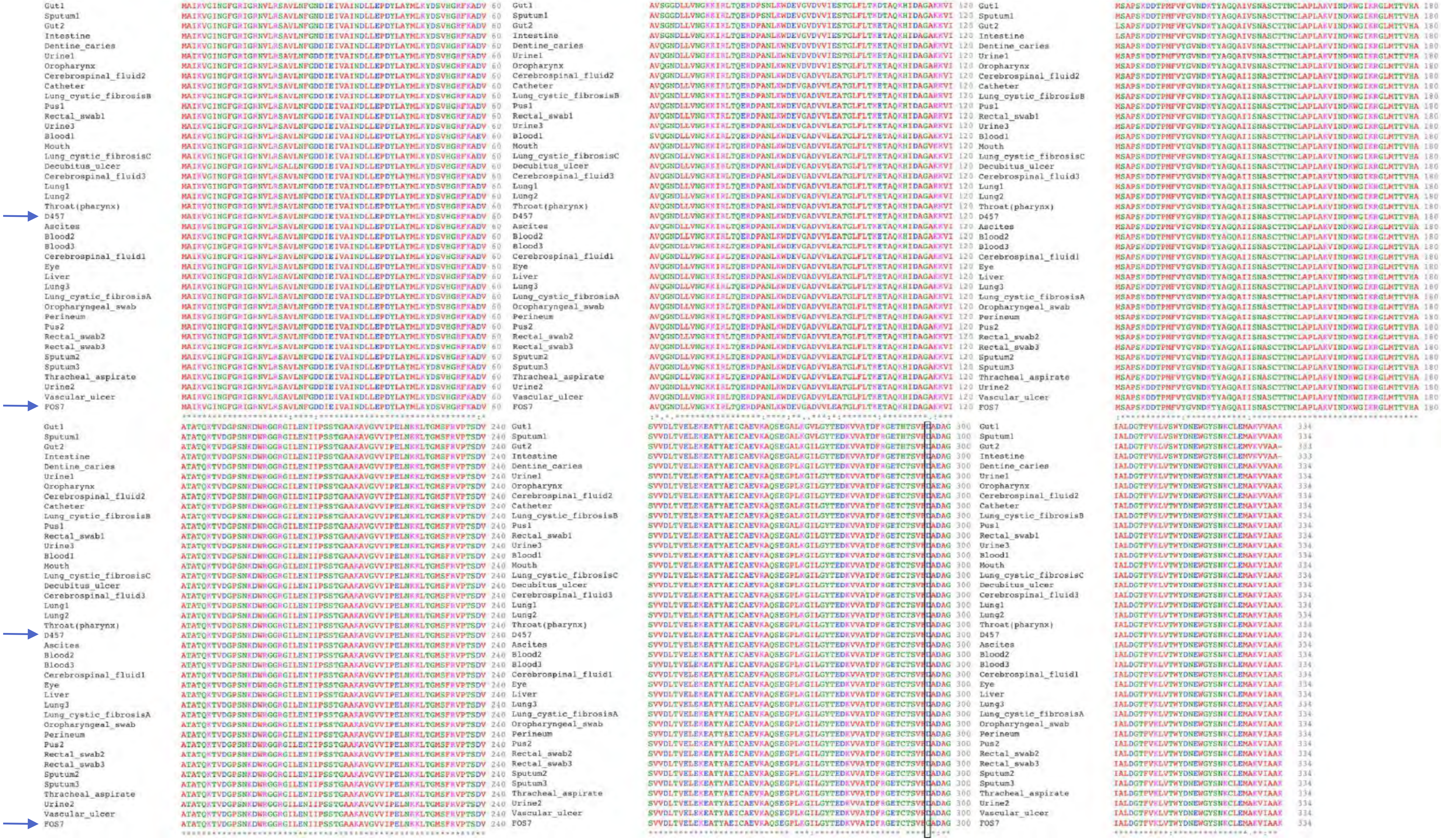

D

Phosphoglycerate kinase

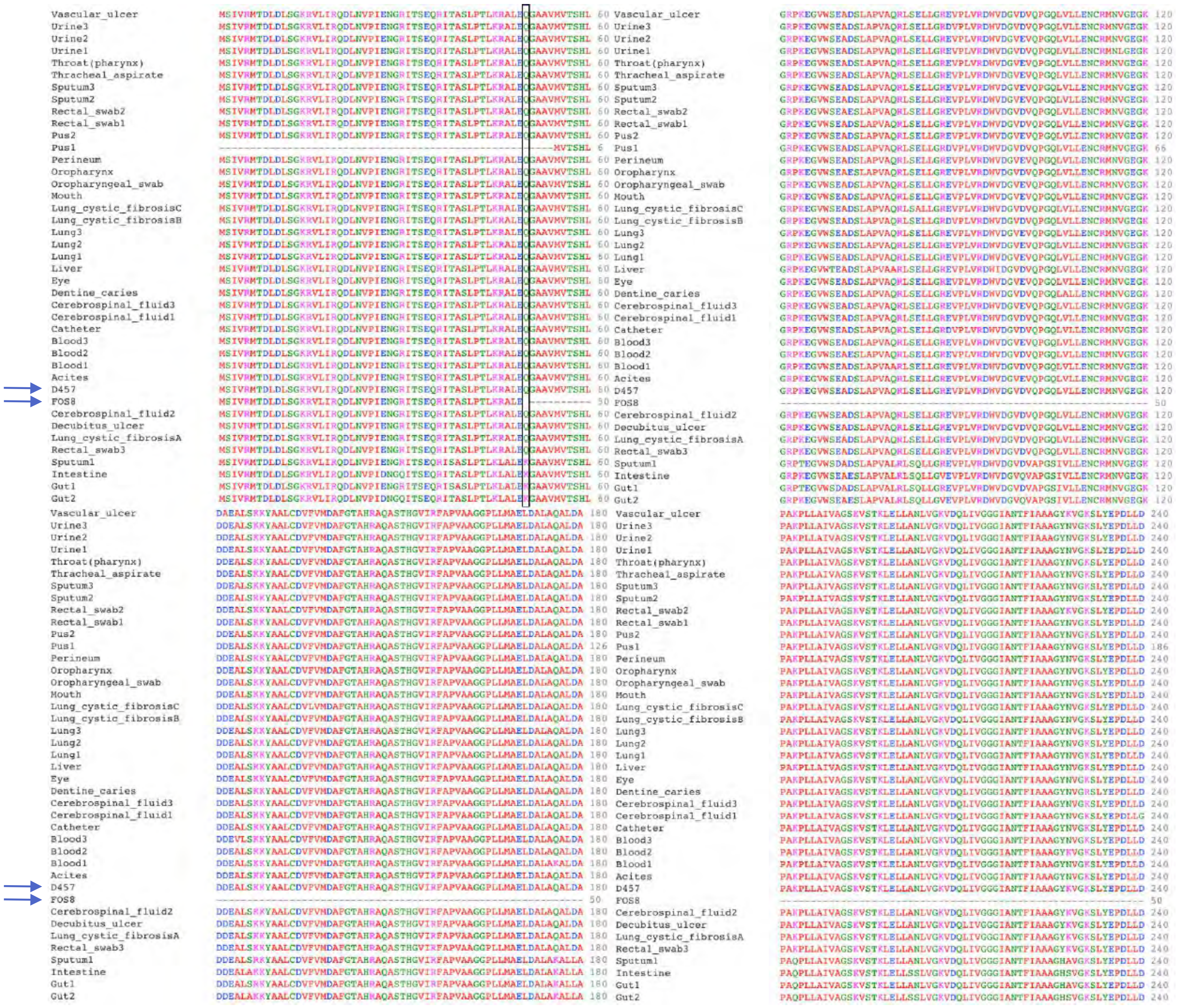

```
Vascular_ulcer              TAKKIVADAKARGADIPLPVDVVTAKQFMPDAVAEVKAVDAVAEDDLILDIGPQTAAQYA 300
Urine3                      TAKKIVADAKARGADIPLPVDVVTAKQFLPDAAAEVKAVDAVAEDDLILDIGPQTAAQYA 300
Urine2                      TAKKIVADAKARGADIPLPVDVVTAKQFMPDAVAEVKAVDAVAEDDLILDIGPQTAAQYA 300
Urine1                      TAKKIVADAKARGADIPLPVDVVTAKQFMPDALAEVKAVDAVAEDDLILDIGPQTAAQYA 300
Throat(pharynx)             TAKKIVADAKARGADIPLPVDVVTAKQFMPDAVAEVKAVDAVAEDDLILDIGPQTAAQYA 300
Thracheal_aspirate          TAKKIVADAKARGADIPLPVDVVTAKQFMPDAVAEVKAVDAVAEDDLILDIGPQTAAQYA 300
Sputum3                     TAKKIVADAKARGADIPLPVDVVTAKQFMPDAVAEVKAVDAVAEDDLILDIGPQTAAQYA 300
Sputum2                     TAKKIVADAKARGADIPLPVDVVTAKQFMPDAVAEVKAVDAVAEDDLILDIGPQTAAQYA 300
Rectal_swab2                TAKKIVADAKARGADIPLPVDVVTAKQFMPDAVAEVKAVDAVAEDDLILDIGPQTAAQYA 300
Rectal_swab1                TAKKIVADAKARGADIPLPVDVVTAKQFLPDAAAEVKAVDAVAEDDLILDIGPQTAAQYA 300
Pus2                        TAKKIVADAKARGADIPLPVDVVTAKQFMPDAVAEVKAVDAVAEDDLILDIGPQTAAQYA 300
Pus1                        TAKKIVADAKARGADIPLPVDVVTAKQFLPDAAAEVKAVDAVAEDDLILDIGPQTAAQYA 246
Perineum                    TAKKIVADAKARGADIPLPVDVVTAKQFMPDAVAEVKAVDAVAEDDLILDIGPQTAAQYA 300
Oropharynx                  TAKKIVADAKARGADIPLPVDVVTAKQFMPDAVAEVKAVDAVAEDDLILDIGPQTAAQYA 300
Oropharyngeal_swab          TAKKIVADAKARGADIPLPVDVVTAKQFMPDAVAEVKAVDAVAEDDLILDIGPQTAAQYA 300
Mouth                       TAKKIVADAKARGADIPLPVDVVTAKQFMPDAVAEVKAVDAVAEDDLILDIGPQTAAQYA 300
Lung_cystic_fibrosisC       TAKKIVADAKARGADIPLPVDVVTAKQFMPDAVAEVKAVDAVAEDDLILDIGPQTAAQYA 300
Lung_cystic_fibrosisB       TAKKIVADAKARGADIPLPVDVVTAKQFLPDAAAEVKAVDAVAEDDLILDIGPQTAAQYA 300
Lung3                       TAKKIVADAKARGADIPLPVDVVTAKQFMPDAVAEVKAVDAVAEDDLILDIGPQTAAQYA 300
Lung2                       TAKKIVADAKARGADIPLPVDVVTAKQFMPDAVAEVKAVDAVAEDDLILDIGPQTAAQYA 300
Lung1                       TAKKIVADAKARGADIPLPVDVVTAKQFMPDAVAEVKAVDAVAEDDLILDIGPQTAAQYA 300
Liver                       TAKKIVADAKARGADIPLPVDVVTAKQFLPDAVAEVKAVDAVAEDDLILDIGPQTAAQYA 300
Eye                         TAKKIVADAKARGADIPLPVDVVTAKQFMPDAVAEVKAVDAVAEDDLILDIGPQTAAQYA 300
Dentine_caries              TAKKIVADAKARGADIPLPVDVVTAKQFMPDAVAEVKAVDAVAEDDLILDIGPQTAAQYA 300
Cerebrospinal_fluid3        TAKKIVADAKARGADIPLPVDVVTAKQFMPDAVAEVKAVDAVAEDDLILDIGPQTAAQYA 300
Cerebrospinal_fluid1        TAKKIVADAKARGADIPLPVDVVTAKQFMPDAVAEVKAVDAVAEDDLILDIGPQTAAQYA 300
Catheter                    TAKKIVADAKARGADIPLPVDVVTAKQFLPDAAAEVKAVDAVAEDDLILDIGPQTAAQYA 300
Blood3                      TAKKIVADAKARGADIPLPVDVVTAKQFMPDAVAEVKAVDAVAEDDLILDIGPQTAAQYA 300
Blood2                      TAKKIVADAKARGADIPLPVDVVTAKQFMPDAVAEVKAVDAVAEDDLILDIGPQTAAQYA 300
Blood1                      TAKKIVADAKARGADIPLPVDVVTAKQFLPDAVAEVKAVDAVAEDDLILDIGPQTAAQYA 300
Acites                      TAKKIVADAKARGADIPLPVDVVTAKQFMPDAVAEVKAVDAVAEDDLILDIGPQTAAQYA 300
D457                        TAKKIVADAKARGADIPLPVDVVTAKQFMPDAVAEVKAVDAVAEDDLILDIGPQTAAQYA 300
FOS8                        ------------------------------------------------------------ 50
Cerebrospinal_fluid2        TAKKIVADAKARGADIPLPVDVVTAKQFMPDAVAEVKAVDAVAEDDLILDIGPQTAAQYA 300
Decubitus_ulcer             TAKKIVADAKARGADIPLPVDVVTAKQFMPDAVAEVKAVDAVAEDDLILDIGPQTAAQYA 300
Lung_cystic_fibrosisA       TAKKIVADAKARGADIPLPVDVVTAKQFMPDAVAEVKAVDAVAEDDLILDIGPQTAAQYA 300
Rectal_swab3                TAKKIVADAKARGADIPLPVDVVTAKQFMPDAVAEVKAVDAVAEDDLILDIGPQTAAQYA 300
Sputum1                     TARRIVADAKARGAEIPLPTDVVVAKQFLPDAEATVKAVTDVAEDDLILDIGPDTAQHYA 300
Intestine                   TAKKIVADAKARGADIPLPTDVVVAKQFLPDAEATVKAVADVAEDDLILDIGPDTAQHYA 300
Gut1                        TARRIVADAKARGAEIPLPTDVVVAKQFLPDAEATVKAVTDVAEDDLILDIGPDTAQHYA 300
Gut2                        TAKKIVADAKARGADIPLPTDVVVAKQFLPDAEATVKAVADVAEDDLILDIGPDTAQHYA 300

Vascular_ulcer              QLIGKAGTVVWNGPVGVFEFEAFSKGTEALARAIASSPAFSIAGGGDTLAAVDKFDIAGQ 360
Urine3                      QLIEKAGTVVWNGPVGVFEFESFSKGTEALARAIASSKAFSIAGGGDTLAAVDKFDIAGD 360
Urine2                      QLIDKAGTVVWNGPVGVFEFEAFSKGTEALARAIASSKAFSIAGGGDTLAAVDKFDIAQQ 360
Urine1                      QLIGKAGTVVWNGPVGVFEFEAFSKGTEALARAIASSPAFSIAGGGDTLAAVDKFDIAQQ 360
Throat(pharynx)             QLIEKAGTVVWNGPVGVFEFEAFSKGTEALARAIASSKAFSIAGGGDTLAAVDKFDIAQQ 360
Thracheal_aspirate          QLIEKAGTVVWNGPVGVFEFEAFSKGTEALARAIASSRAFSIAGGGDTLAAVDKFDIAQQ 360
Sputum3                     QLIDKAGTVVWNGPVGVFEFEAFSKGTEALARAIASSRAFSIAGGGDTLAAVDKFDIAQQ 360
Sputum2                     QLIDKAGTVVWNGPVGVFEFEAFSKGTEALARAIASSKAFSIAGGGDTLAAVDKFDIAQQ 360
Rectal_swab2                QLIGKAGTVVWNGPVGVFEFEAFSKGTEALARAIASSPAFSIAGGGDTLAAVDKFDIAGQ 360
Rectal_swab1                QLIENAGTVVWNGPVGVFEFEAFSKGTEALARAIASSKAFSIAGGGDTLAAVDKFDIAKD 360
Pus2                        QLIDKAGTVVWNGPVGVFEFEAFSKGTEALARAIASSRAFSIAGGGDTLAAVDKFDIAQQ 360
Pus1                        QLIEKAGTVVWNGPVGVFEFESFSKGTEALARAIASSKAFSIAGGGDTLAAVDKFDIAGD 306
Perineum                    QLIDKAGTVVWNGPVGVFEFEAFSKGTEALARAIASSKAFSIAGGGDTLAAVDKFDIAQQ 360
Oropharynx                  QLIDKAGTVVWNGPVGVFEFEAFSKGTEALARAIASSPAFSIAGGGDTLAAVDKFDIAQQ 360
Oropharyngeal_swab          QLIDKAGTVVWNGPVGVFEFEAFSKGTEALARAIASSKAFSIAGGGDTLAAVDKFDIAQQ 360
Mouth                       QLIDKAGTVVWNGPVGVFEFEAFSKGTEALARAIASSKAFSIAGGGDTLAAVDKFDIAQQ 360
Lung_cystic_fibrosisC       QLIDKAGTVVWNGPVGVFEFDAFSKGTEALARAIAGSKAFSIAGGGDTLAAVDKFDIAGQ 360
Lung_cystic_fibrosisB       QLIEKAGTVVWNGPVGVFEFESFSKGTEALARAIASSKAFSIAGGGDTLAAVDKFDIAGD 360
Lung3                       QLIEKAGTVVWNGPVGVFEFEAFSKGTEALARAIASSKAFSIAGGGDTLAAVDKFDIAQQ 360
Lung2                       QLIEKAGTVVWNGPVGVFEFEAFSKGTEALARAIASSKAFSIAGGGDTLAAVDKFDIAQQ 360
Lung1                       QLIEKAGTVVWNGPVGVFEFEAFSKGTEALARAIASSKAFSIAGGGDTLAAVDKFDIAQQ 360
Liver                       QLIEKAGTVVWNGPVGVFEFEAFSKGTEALARAIASSPAFSIAGGGDTLAAVDKFDIAKD 360
Eye                         QLIEKAGTVVWNGPVGVFEFEAFSKGTEALARAIASSKAFSIAGGGDTLAAVDKFDIAQQ 360
Dentine_caries              QLIGKAGTVVWNGPVGVFEFEAFSKGTEALARAIASSPAFSIAGGGDTLAAVDKFDIAQQ 360
Cerebrospinal_fluid3        QLIEKAGTVVWNGPVGVFEFEAFSKGTEALARAIASSKAFSIAGGGDTLAAVDKFDIAQQ 360
Cerebrospinal_fluid1        QLIEKAGTVVWNGPVGVFEFEAFSKGTEALARAIASSPAFSIAGGGDTLAAVDKFDIAKD 360
Catheter                    QLIEKAGTVVWNGPVGVFEFESFSKGTEALARAIASSKAFSIAGGGDTLAAVDKFDIAGD 360
Blood3                      QLIDKAGTVVWNGPVGVFEFEAFSKGTEALARAIASSRAFSIAGGGDTLAAVDKFDIAQQ 360
Blood2                      QLIGKAGTVVWNGPVGVFEFEAFSKGTEALARAIASSPAFSIAGGGDTLAAVDKFDIAGQ 360
Blood1                      QLIEKAGTVVWNGPVGVFEFEAFSKGTEALARAIASSKAFSIAGGGDTLAAVDKFDIAGD 360
Acites                      QLIEKAGTVVWNGPVGVFEFEAFSKGTEALARAIASSKAFSIAGGGDTLAAVDKFDIAQQ 360
D457                        QLIGKAGTVVWNGPVGVFEFEAFSKGTEALARAIASSPAFSIAGGGDTLAAVDKFDIAGQ 360
FOS8                        ------------------------------------------------------------ 50
Cerebrospinal_fluid2        QLIGKAGTVVWNGPVGVFEFEAFSKGTEALARAIASSPAFSIAGGGDTLAAVDKFDIAGQ 360
Decubitus_ulcer             QLIGKAGTVVWNGPVGVFEFEAFSKGTEALARAIASSPAFSIAGGGDTLAAVDKFDIAGQ 360
Lung_cystic_fibrosisA       QLIGKAGTVVWNGPVGVFEFEAFSKGTEALARAIASSPAFSIAGGGDTLAAVDKFDIAGQ 360
Rectal_swab3                QLIGKAGTVVWNGPVGVFEFEAFSKGTEALARAIASSPAFSIAGGGDTLAAVDKFDIAGQ 360
Sputum1                     GLIAKAGTVVWNGPVGVFEFEAFSHGTQALAQAIAASSAFSIAGGGDTLAAVDKYDIADQ 360
Intestine                   GLIAKAGTVVWNGPVGVFEFEAFSHGTEALARAIAASPAFSIAGGGDTLAAVDKYDIAGE 360
Gut1                        GLIAKAGTVVWNGPVGVFEFEAFSHGTQALAQAIAASSAFSIAGGGDTLAAVDKYDIADQ 360
Gut2                        GLIAKAGTVVWNGPVGVFEFEAFSHGTEALARAIAASPAFSIAGGGDTLAAVDKYDIAGE 360

Vascular_ulcer              VSYISTGGGAFLEFLEGKTLPAVAALDARGA      391
Urine3                      VSYISTGGGAFLEFLEGKTLPAVAALDARGA      391
Urine2                      VSYISTGGGAFLEFLEGKTLPAVAALDARGA      391
Urine1                      VSYISTGGGAFLEFLEGKTLPAVAALDARGA      391
Throat(pharynx)             VSYISTGGGAFLEFLEGKTLPAVAALDARGA      391
Thracheal_aspirate          VSYISTGGGAFLEFLEGKTLPAVAALDARGA      391
Sputum3                     VSYISTGGGAFLEFLEGKTLPAVAALDARGA      391
Sputum2                     VSYISTGGGAFLEFLEGKTLPAVAALDARGA      391
Rectal_swab2                VSYISTGGGAFLEFLEGKTLPAVAALDARGA      391
Rectal_swab1                VSYISTGGGAFLEFLEGKTLPAVAALDARGA      391
Pus2                        VSYISTGGGAFLEFLEGKTLPAVAALDARGA      391
Pus1                        VSYISTGGGAFLEFLE---------------      322
Perineum                    VSYISTGGGAFLEFLEGKTLPAVAALDARGA      391
Oropharynx                  VSYISTGGGAFLEFLEGKTLPAVAALDARGA      391
Oropharyngeal_swab          VSYISTGGGAFLEFLEGKTLPAVAALDARGA      391
Mouth                       VSYISTGGGAFLEFLEGKTLPAVAALDARGA      391
Lung_cystic_fibrosisC       VSYISTGGGAFLEFLEGKTLPAVAALDARGA      391
Lung_cystic_fibrosisB       VSYISTGGGAFLEFLEGKTLPAVAALDARGA      391
Lung3                       VSYISTGGGAFLEFLEGKTLPAVAALDARGA      391
Lung2                       VSYISTGGGAFLEFLEGKTLPAVAALDARGA      391
Lung1                       VSYISTGGGAFLEFLEGKTLPAVAALDARGA      391
Liver                       VSYISTGGGAFLEFLEGKTLPAVAALDARGA      391
Eye                         VSYISTGGGAFLEFLEGKTLPAVAALDARGA      391
Dentine_caries              VSYISTGGGAFLEFLEGKTLPAVAALDARGA      391
Cerebrospinal_fluid3        VSYISTGGGAFLEFLEGKTLPAVAALDARGA      391
Cerebrospinal_fluid1        VSYISTGGGAFLEFLEGKTLPAVAALDARGA      391
Catheter                    VSYISTGGGAFLEFLEGKTLPAVAALDARGA      391
Blood3                      VSYISTGGGAFLEFLEGKTLPAVAALDARGA      391
Blood2                      VSYISTGGGAFLEFLEGKTLPAVAALDARGA      391
Blood1                      VSYISTGGGAFLEFLEGKTLPAVAALDARGA      391
Acites                      VSYISTGGGAFLEFLEGKTLPAVAALDARGA      391
D457                        VSYISTGGGAFLEFLEGKTLPAVAALDARGA      391
FOS8                        -------------------------------      50
Cerebrospinal_fluid2        VSYISTGGGAFLEFLEGKTLPAVAALDARGA      391
Decubitus_ulcer             VSYISTGGGAFLEFLEGKTLPAVAALDARGA      391
Lung_cystic_fibrosisA       VSYISTGGGAFLEFLEGKTLPAVAALDARGA      391
Rectal_swab3                VSYISTGGGAFLEFLEGKTLPAVAALDARGA      391
Sputum1                     VSYISTGGGAFLEFLEGKTLPAVAALEARGQ      391
Intestine                   VSYISTGGGAFLEFLEGKTLPAVAALEARGS      391
Gut1                        VSYISTGGGAFLEFLEGKTLPAVAALEARGQ      391
Gut2                        VSYISTGGGAFLEFLEGKTLPAVAALEARGS      391
```
