# Supplementary figures and images for: The Inactivation of Enzymes Belonging to the Central Carbon Metabolism Is a Novel Mechanism of Developing Antibiotic Resistance
Source: mSystems. 2020 Jun 2;5(3):e00282-20. doi: 10.1128/mSystems.00282-20 (PMC8534728; doi:10.1128/mSystems.00282-20)

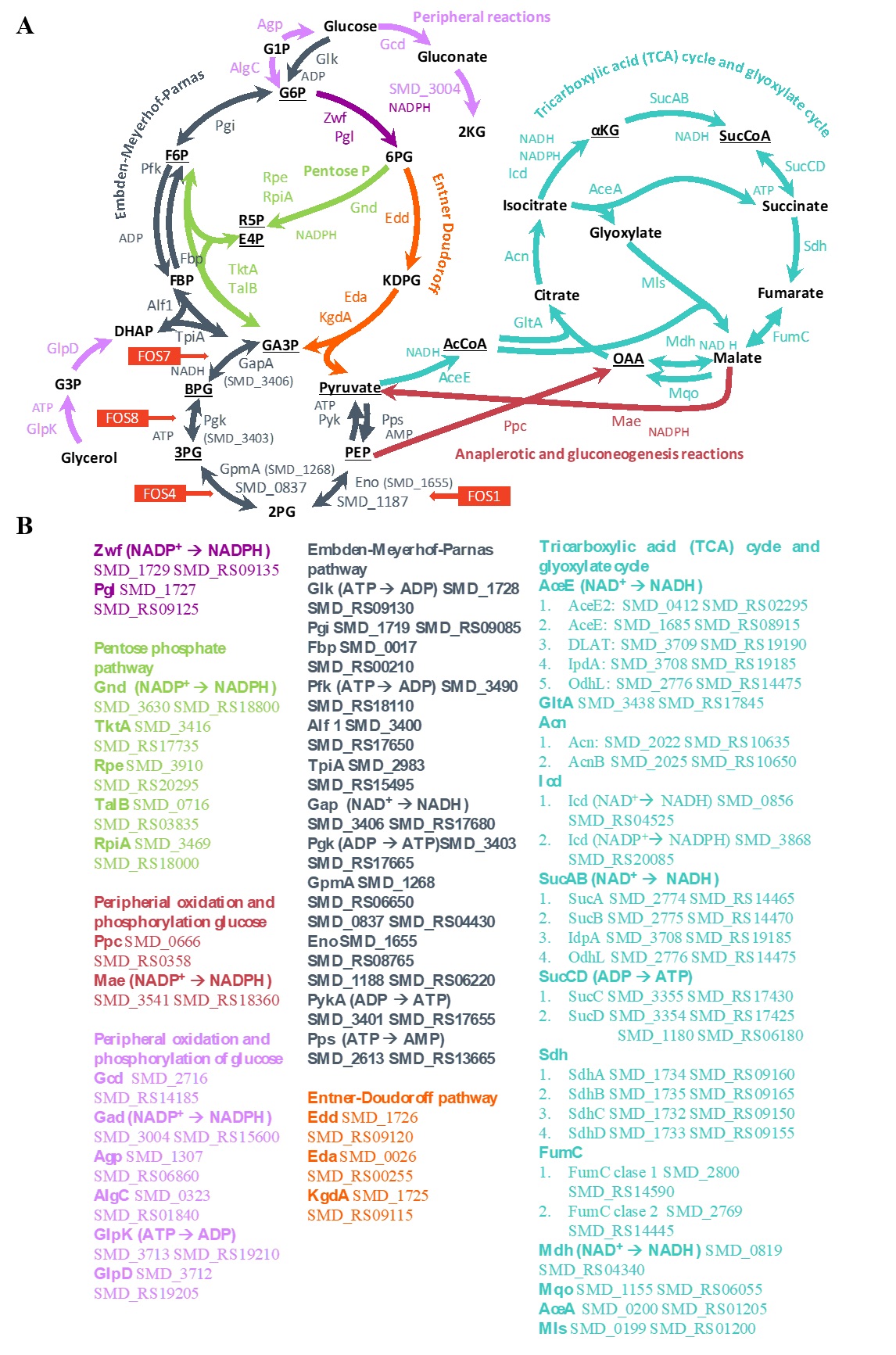

Supplement: FIG S3 [file msystems.00282-20-sf003.jpg]

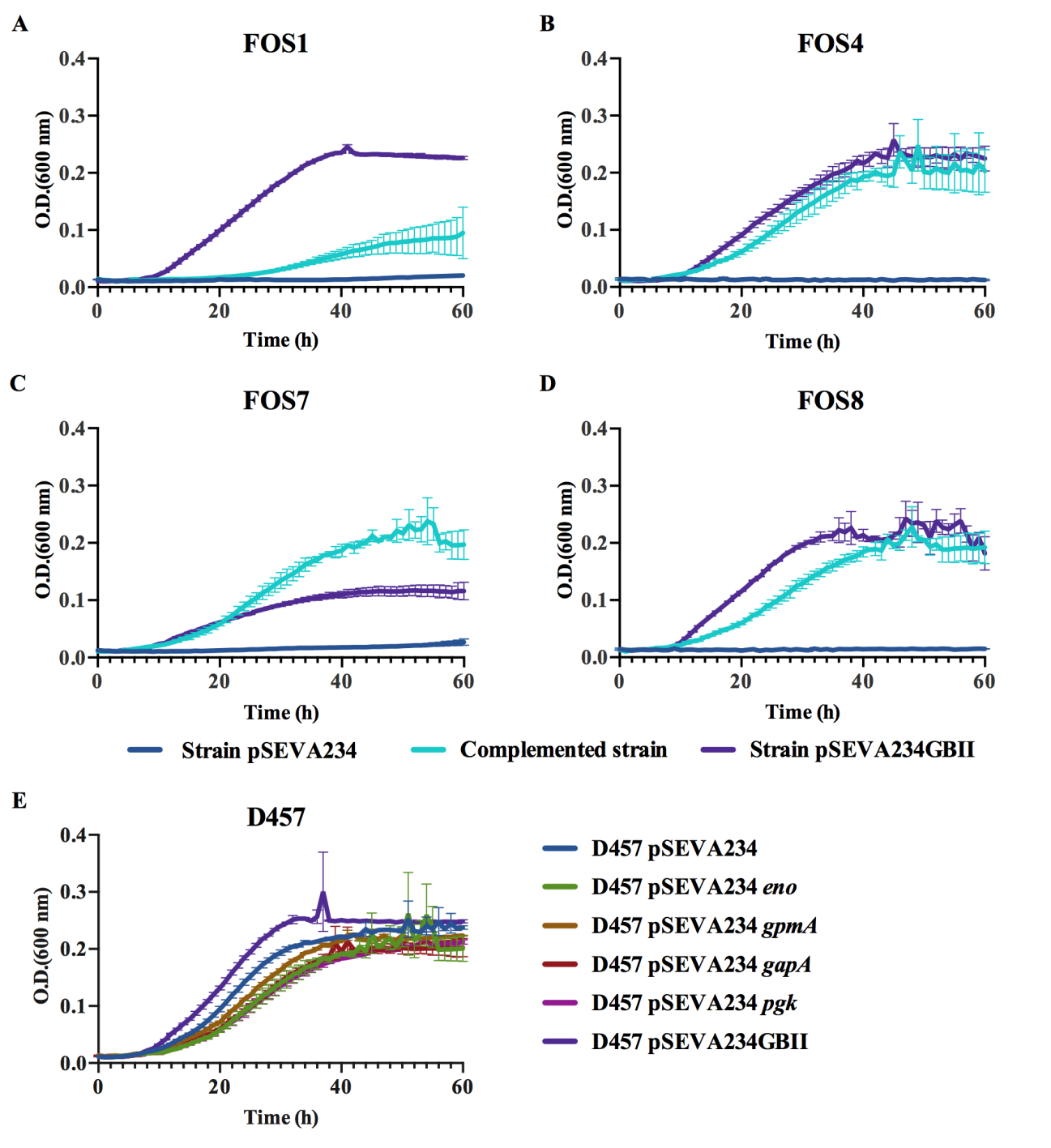

Supplement: FIG S4 [file msystems.00282-20-sf004.tif]

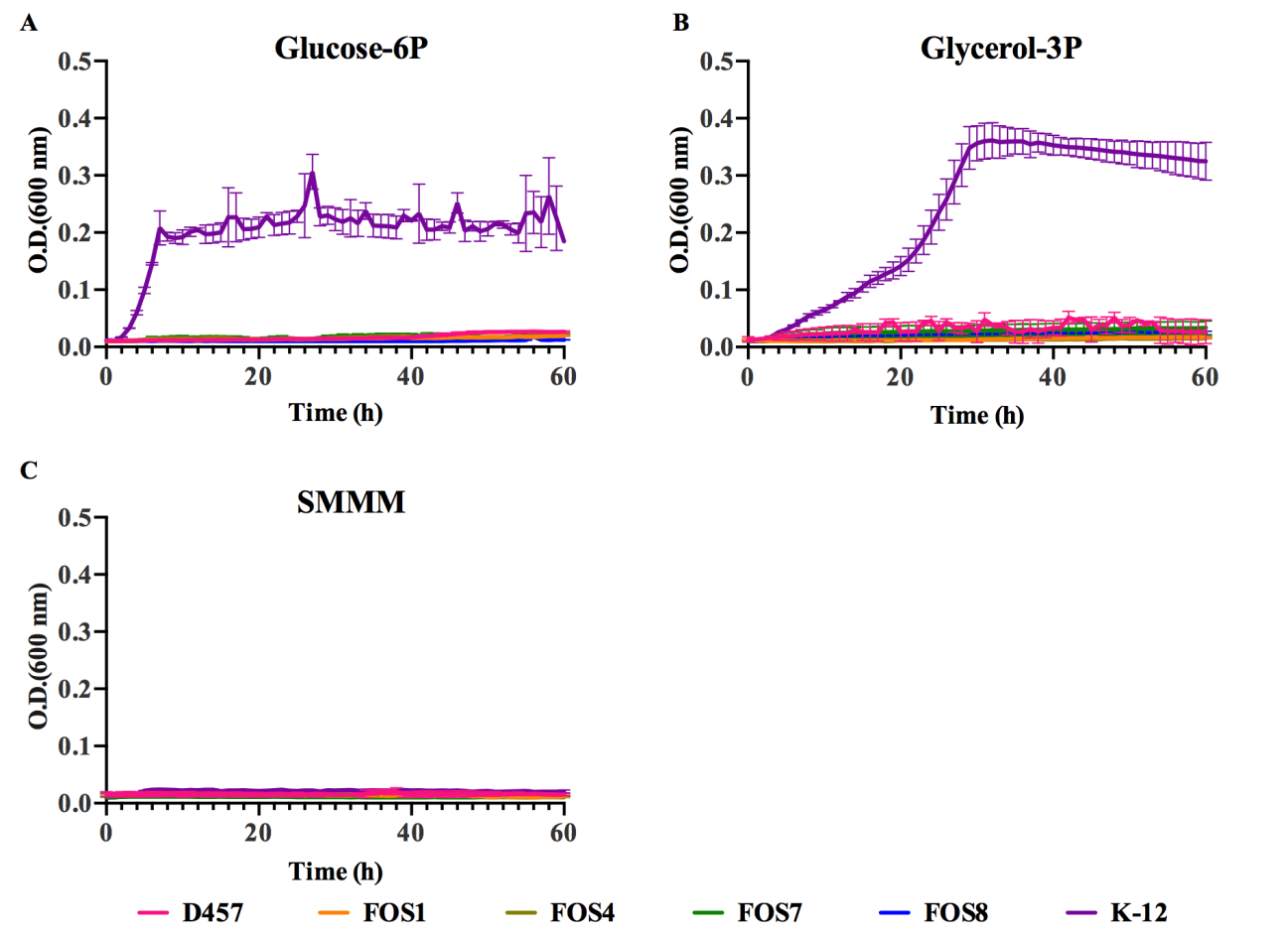

Supplement: FIG S5 [file msystems.00282-20-sf005.tif]
