# Supplementary material for: The Inactivation of Enzymes Belonging to the Central Carbon Metabolism Is a Novel Mechanism of Developing Antibiotic Resistance
Source: mSystems. 2020 Jun 2;5(3):e00282-20. doi: 10.1128/mSystems.00282-20 (PMC8534728; doi:10.1128/mSystems.00282-20)
Supplement: TABLE S1 [file msystems.00282-20-st001.docx]

**Table S1. RPKM and fold change (log_2_) of genes presenting different level of expression in the mutant strains in comparison with the wild-type D457 strain**

| **RPKM FOLD CHANGE (log_2_)**  **ID – Gene D457 FOS1 FOS4 FOS7 FOS8 FOS1 FOS4 FOS7 FOS8** | | | | | | | | | |
| --- | --- | --- | --- | --- | --- | --- | --- | --- | --- |
| SMD_0241 | 19 | 18 | 19 | 6 | 9 | -0.08 | 0.00 | **-1.66** | **-1.08** |
| SMD_0337 16SrRNA | 754 | 761 | 632 | 115 | 521 | 0.01 | -0.25 | **-2.71** | -0.53 |
| SMD_0340 23SrRNA | 968 | 904 | 691 | 270 | 696 | -0.10 | -0.49 | **-1.84** | -0.48 |
| SMD_0342 16SrRNA | 597 | 608 | 505 | 92 | 416 | 0.03 | -0.24 | **-2.70** | -0.52 |
| SMD_0345 23SrRNA | 961 | 883 | 691 | 260 | 695 | -0.12 | -0.48 | **-1.89** | -0.47 |
| SMD_0365 | 72 | 36 | 56 | 74 | 85 | **-1.00** | -0.36 | 0.04 | 0.24 |
| SMD_0475 | 192 | 94 | 189 | 250 | 252 | **-1.03** | -0.02 | 0.38 | 0.39 |
| SMD_0480 | 8 | 8 | 7 | 6 | 16 | 0.00 | -0.19 | -0.42 | **1.00** |
| SMD_0594 | 11 | 16 | 6 | 18 | 29 | 0.54 | -0.87 | 0.71 | **1.40** |
| SMD_1067 | 11 | 7 | 14 | 23 | 14 | BT | BT | **1.06** | BT |
| SMD_1071 | 27 | 15 | 46 | 66 | 33 | -0.85 | 0.77 | **1.29** | 0.29 |
| SMD_1154 | 10 | 8 | 20 | 21 | 10 | BT | **1.00** | **1.07** | BT |
| SMD_1156 | 51 | 22 | 47 | 61 | 60 | **-1.21** | -0.12 | 0.26 | 0.23 |
| SMD_1210 *tRNA-Thr* | 6 | 16 | 17 | 19 | 14 | **1.42** | **1.50** | **1.66** | BT |
| SMD_1276 *creD* | 7 | 8 | 18 | 10 | 11 | BT | **1.36** | BT | BT |
| SMD_1277 | 7 | 7 | 18 | 13 | 15 | BT | **1.36** | BT | **1.10** |
| SMD_1355 *bfr3* | 386 | 563 | 232 | 175 | 275 | 0.54 | -0.73 | **-1.14** | -0.49 |
| SMD_1423 | 17 | 14 | 8 | 8 | 13 | -0.28 | **-1.09** | **-1.09** | -0.39 |
| SMD_1691 | 71 | 67 | 46 | 31 | 33 | -0.08 | -0.63 | **-1.20** | **-1.11** |
| SMD_1728 *glk* | 100 | 97 | 49 | 61 | 81 | -0.04 | **-1.03** | -0.71 | -0.30 |
| SMD_2107 | 42 | 26 | 48 | 44 | 19 | -0.69 | 0.19 | 0.07 | **-1.14** |
| SMD_2119 *iroE* | 12 | 8 | 20 | 24 | 14 | BT | 0.74 | **1.00** | BT |
| SMD_2221 | 94 | 75 | 58 | 42 | 40 | -0.33 | -0.70 | **-1.16** | **-1.23** |
| SMD_2227 *fruA* | 151 | 101 | 125 | 92 | 62 | -0.58 | -0.27 | -0.71 | **-1.28** |
| SMD_2228 *rpfN* | 195 | 133 | 166 | 114 | 77 | -0.55 | -0.23 | -0.77 | **-1.34** |
| SMD_2330 | 21 | 9 | 18 | 23 | 19 | **-1.22** | -0.22 | 0.13 | -0.14 |
| SMD_2470 | 6 | 11 | 14 | 16 | 10 | BT | BT | **1.42** | BT |
| SMD_2486 nrdF | 26 | 28 | 9 | 9 | 13 | 0.11 | **-1.53** | **-1.53** | **-1.00** |
| SMD_2487 *nrdE* | 27 | 39 | 8 | 11 | 19 | 0.53 | **-1.75** | **-1.30** | -0.51 |
| SMD_2491 | 333 | 471 | 89 | 105 | 195 | 0.50 | **-1.90** | **-1.67** | -0.77 |
| SMD_2492 *mdtI* | 259 | 355 | 46 | 78 | 147 | 0.45 | **-2.49** | **-1.73** | -0.82 |
| SMD_2493 | 340 | 398 | 66 | 100 | 214 | 0.23 | **-2.36** | **-1.77** | -0.67 |
| SMD_2575 | 41 | 50 | 21 | 16 | 16 | 0.29 | -0.97 | **-1.36** | **-1.36** |
| SMD_2576 | 89 | 94 | 36 | 33 | 43 | 0.08 | **-1.31** | **-1.43** | **-1.05** |
| SMD_2577 *adi* | 241 | 252 | 80 | 60 | 86 | 0.06 | **-1.59** | **-2.01** | **-1.49** |
| SMD_2578 | 312 | 345 | 132 | 90 | 143 | 0.15 | **-1.24** | **-1.79** | **-1.13** |
| SMD_2691 *hutH* | 107 | 130 | 26 | 62 | 105 | 0.28 | **-2.04** | -0.79 | -0.03 |
| SMD_2692 | 87 | 97 | 24 | 37 | 66 | 0.16 | **-1.86** | **-1.23** | -0.40 |
| SMD_2693 | 76 | 98 | 35 | 55 | 72 | 0.37 | **-1.12** | -0.47 | -0.08 |
| SMD_2853 *cydC* | 90 | 58 | 42 | 42 | 55 | -0.63 | **-1.10** | **-1.10** | -0.71 |
| SMD_2856 *cydB* | 907 | 972 | 536 | 423 | 545 | 0.10 | -0.76 | **-1.10** | -0.73 |
| SMD_2857 | 1608 | 1969 | 946 | 607 | 888 | 0.29 | -0.77 | **-1.41** | -0.86 |
| SMD_2858  *tRNA-Pro* | 439 | 511 | 289 | 195 | 241 | 0.22 | -0.60 | **-1.17** | -0.87 |
| SMD_2881 *yidD* | 79 | 38 | 57 | 67 | 93 | **-1.06** | -0.47 | -0.24 | 0.24 |
| SMD_3108 | 26 | 21 | 23 | 60 | 45 | -0.31 | -0.18 | **1.21** | 0.79 |
| SMD_3161 | 24 | 23 | 38 | 51 | 37 | -0.06 | 0.66 | **1.09** | 0.62 |
| SMD_3348 | 19 | 9 | 17 | 23 | 24 | **-1.08** | -0.16 | 0.28 | 0.34 |
| SMD_3402 *gph2* | 146 | 157 | 170 | 157 | 42 | 0.10 | 0.22 | 0.10 | **-1.80** |
| SMD_3410 | 26 | 35 | 13 | 13 | 26 | 0.43 | **-1.00** | **-1.00** | 0.00 |
| SMD_3411 | 18 | 30 | 9 | 15 | 19 | 0.74 | **-1.00** | -0.26 | 0.08 |
| SMD_3412 *blaI* | 9 | 19 | 12 | 16 | 16 | **1.08** | BT | 0.83 | 0.83 |
| SMD_3450 *selA* | 41 | 38 | 18 | 23 | 34 | -0.11 | **-1.19** | -0.83 | -0.27 |
| SMD_3475 | 31 | 37 | 26 | 15 | 25 | 0.26 | -0.25 | **-1.05** | -0.31 |
| SMD_3502 *rpoE2* | 12 | 11 | 33 | 30 | 15 | BT | **1.46** | **1.32** | 0.32 |
| SMD_3549 | 180 | 111 | 135 | 161 | 34 | -0.70 | -0.42 | -0.16 | **-2.40** |
| SMD_3550 *citM* | 120 | 83 | 109 | 111 | 20 | -0.53 | -0.14 | -0.11 | **-2.58** |
| SMD_3551 *fabG3* | 143 | 81 | 126 | 129 | 31 | -0.82 | -0.18 | -0.15 | **-2.21** |
| SMD_3614 | 106 | 99 | 48 | 49 | 73 | -0.10 | **-1.14** | **-1.11** | -0.54 |
| SMD_3757 | 16 | 10 | 7 | 10 | 12 | -0.68 | **-1.19** | -0.68 | -0.42 |
| SMD_3802 | 50 | 22 | 61 | 55 | 32 | **-1.18** | 0.29 | 0.14 | -0.64 |
| SMD_4179 23SrRNA | 968 | 904 | 694 | 274 | 703 | -0.10 | -0.48 | **-1.82** | -0.46 |
| SMD_4182 16SrRNA | 681 | 694 | 588 | 108 | 477 | 0.03 | -0.21 | **-2.66** | -0.51 |
| SMD_4184 23SrRNA | 968 | 901 | 693 | 269 | 696 | -0.10 | -0.48 | **-1.85** | -0.48 |
| SMD_4187 16SrRNA | 672 | 686 | 592 | 106 | 468 | 0.03 | -0.18 | **-2.66** | -0.52 |

BT. Below threshold. Only relevant fold changes, (log_2_ <-1 or > 1), in any of the samples are included. Bold: fold changes (log_2_) <-1 or > 1.
