# Supplementary material for: The Inactivation of Enzymes Belonging to the Central Carbon Metabolism Is a Novel Mechanism of Developing Antibiotic Resistance
Source: mSystems. 2020 Jun 2;5(3):e00282-20. doi: 10.1128/mSystems.00282-20 (PMC8534728; doi:10.1128/mSystems.00282-20)
Supplement: TABLE S2 [file msystems.00282-20-st002.docx]

**Table S2. Bacterial strains** **and plasmids used in this work**

| **Strain** | **Relevant characteristics** | **Reference or source** |
| --- | --- | --- |
| *E. coli* |  |  |
| CC118λpir | Cloning host. Δ(*ara-leu*), *ara*D, Δ*lac*x74, *gal*E, *gal*K, *pho*A20, *thi*-1, *rps*E, *rpo*B, *arg*E (Am), *rec*A1, *λpir lysogen* | (1) |
| One Shot™ OmniMAX™ | F´ {proAB lacIq lacZΔM15 Tn10(TetR) Δ(ccdAB) mcrA Δ(mrr hsdRMS-mcrBC) Φ 80(lacZ)ΔM15 Δ(lacZYA-argF)U169 endA1 recA1 supE44 thi-1 gyrA96 relA1 tonA panD | Invitrogen, Life Technologies |
| 1047 (pRK2013) | Conjugation helper. pRK2013 (Kan^R^) | (2) |
| BW25113 | *E. coli* K-12 BD792 F-, λ- | (3) |
| JW3641 | Deletion mutant *uhpT* | (4) |
| ALB02 | CC118λpir pSEVA234 | This study |
| TGG01 (CC118λpir pGEMT *eno*) | CC118λpir with pTGG01 | This study |
| ALB03 (CC118λpir pGEMT *gpmA*) | CC118λpir with pBA04 | This study |
| ALB04 (CC118λpir pGEMT *pgk*) | CC118λpir with pBA05 | This study |
| ALB05 (CC118λpir pGEMT *gapA*) | CC118λpir with pBA06 | This study |
| TGG02 (CC118λpir pSEVA234 *eno*) | CC118λpir with pTGG02 | This study |
| TGG03 (CC118λpir pSEVA234 GapA-Pgk- GpmA- Eno) | CC118λpir with pTGG03 | This study |
| TGG04 (CC118λpir pGEMT *Zwf*AB) | CC118λpir with pTGG04 | This study |
| TGG05 (CC118λpir pEx18Tc *Zwf*AB) | CC118λpir with pTGG05 | This study |
| *P. aeruginosa* |  |  |
| PA14 | Burn wound isolate | (5) |
| PA14 GlpT (PA14_MrT7_417wor) | Transposon insertion mutant *glpT* | (5) |
| PA14 FosA (PA14_MrT7_380wor) | Transposon insertion mutant *fosA* | (5) |
| *S. maltophilia* |  |  |
| D457 | Bronchial aspirate isolate | (6) |
| E729 | Urine clinical isolate | (7) |
| E227 | Blood clinical isolate | (7) |
| E759 | Sputum clinical isolate | (7) |
| JLM8 E923 | Sputum clinical isolate | (7) |
| E999 | Respiratory secretion clinical isolate | (7) |
| G51 | Blood clinical isolate | (7) |
| E539 | Pus from a wound clinical isolate | (7) |
| E301 | Urine clinical isolate | (7) |
| D388 | Urine clinical isolate | (7) |
| C048 | Urine clinical isolate | (7) |
| E861 | Sputum clinical isolate | (7) |
| C357 | Urine clinical isolate | (7) |
| F375 | Blood clinical isolate | (7) |
| E824 | Blood clinical isolate | (7) |
| E729 | Urine clinical isolate | (7) |
| E227 | Blood clinical isolate | (7) |
| FOS1 | Fosfomycin resistant mutant | This study |
| FOS4 | Fosfomycin resistant mutant | This study |
| FOS7 | Fosfomycin resistant mutant | This study |
| FOS8 | Fosfomycin resistant mutant | This study |
| ALB06 | D457 with pSEVA234 | This study |
| TGG04 | FOS1 with pSEVA234 | This study |
| ALB07 | FOS4 with pSEVA234 | This study |
| ALB08 | FOS7 with pSEVA234 | This study |
| ALB09 | FOS8 with pSEVA234 | This study |
| TGG05 (D457 pSEVA234 *eno*) | D457 with pTGG02 | This study |
| TGG06 (D457 pSEVA234 *gpmA*) | D457 with pBA07 | This study |
| ALB10 (D457 pSEVA234 *gapA*) | D457 with pBA08 | This study |
| ALB11 (D457 pSEVA234 *pgk*) | D457 with pBA09 | This study |
| TGG07 (FOS1 pSEVA234 *eno*) | FOS1 with pTGG02 | This study |
| ALB12 (FOS4 pSEVA234 *gpmA*) | FOS4 with pBA07 | This study |
| ALB13 (FOS7 pSEVA234 *gapA*) | FOS7 with pBA08 | This study |
| ALB14 (FOS8 pSEVA234 *pgk*) | FOS8 with pBA09 | This study |
| TGG08 (D457 pSEVA234 *gapA*- *pgk*- *gpmA*- *eno*) | D457 with pTGG03 | This study |
| TGG09 (FOS1 pSEVA234 *gapA*- *pgk*- *gpmA*- *eno*) | FOS1 with pTGG03 | This study |
| TGG10 (FOS4 pSEVA234 *gapA*- *pgk*- *gpmA*- *eno*) | FOS4 with pTGG03 | This study |
| TGG11 (FOS7 pSEVA234 *gapA*- *pgk*- *gpmA*- *eno*) | FOS7 with pTGG03 | This study |
| TGG12 (FOS8 pSEVA234 *gapA*- *pgk*- *gpmA*- *eno*) | FOS8 with pTGG03 | This study |
| TGG13 | D457 Δ*zwf* | This study |
| TGG14 | FOS4Δ*zwf* | This study |
| TGG15 | FOS7 Δ*zwf* | This study |
| **Plasmids** | **Relevant characteristics** | **Reference or source** |
| pGEM -T Easy | Cloning plasmid, AmpR | Promega |
| pSEVA234 | KmR; standard SEVA expression vector, oriV(pBBR1) lacIQ, Ptrc | (8) |
| pS224·GBII | KmR; pSEVA224 derivative bearing Module II as a BamHI HindIII insert | (9) |
| pTGG01 | pGEM-T *eno* | This study |
| pBA04 | pGEM-T *gpmA* | This study |
| pBA05 | pGEM-T *pgk* | This study |
| pBA06 | pGEM-T *gapA* | This study |
| pTGG02 | pSEVA234 *eno* | This study |
| pBA07 | pSEVA234 *gpmA* | This study |
| pBA08 | pSEVA234 *gapA* | This study |
| pBA09 | pSEVA234 *pgk* | This study |
| pTGG03 | pSEVA234GBII (*gapA- pgk-gpmA-eno* from *E.coli* K-12) | This study |
| pTGG04 | pGEM-T ZwfAB | This study |
| pTGG05 | pEx18Tc ZwfAB | This study |

1. de Lorenzo V, Timmis KN. 1994. Analysis and construction of stable phenotypes in gram-negative bacteria with Tn5- and Tn10-derived minitransposons. Methods Enzymol 235:386-405.

2. Figurski DH, Helinski DR. 1979. Replication of an origin-containing derivative of plasmid RK2 dependent on a plasmid function provided in trans. Proc Natl Acad Sci U S A 76:1648-52.

3. Datsenko KA, Wanner BL. 2000. One-step inactivation of chromosomal genes in Escherichia coli K-12 using PCR products. Proc Natl Acad Sci U S A 97:6640-5.

4. Baba T, Ara T, Hasegawa M, Takai Y, Okumura Y, Baba M, Datsenko KA, Tomita M, Wanner BL, Mori H. 2006. Construction of Escherichia coli K-12 in-frame, single-gene knockout mutants: the Keio collection. Mol Syst Biol 2:2006 0008.

5. Liberati NT, Urbach JM, Miyata S, Lee DG, Drenkard E, Wu G, Villanueva J, Wei T, Ausubel FM. 2006. An ordered, nonredundant library of Pseudomonas aeruginosa strain PA14 transposon insertion mutants. Proc Natl Acad Sci U S A 103:2833-8.

6. Alonso A, Martinez JL. 1997. Multiple antibiotic resistance in Stenotrophomonas maltophilia. Antimicrob Agents Chemother 41:1140-2.

7. Alonso A, Martinez JL. 2001. Expression of multidrug efflux pump SmeDEF by clinical isolates of Stenotrophomonas maltophilia. Antimicrob Agents Chemother 45:1879-81.

8. Silva-Rocha R, Martinez-Garcia E, Calles B, Chavarria M, Arce-Rodriguez A, de Las Heras A, Paez-Espino AD, Durante-Rodriguez G, Kim J, Nikel PI, Platero R, de Lorenzo V. 2013. The Standard European Vector Architecture (SEVA): a coherent platform for the analysis and deployment of complex prokaryotic phenotypes. Nucleic Acids Res 41:D666-75.

9. Sanchez-Pascuala A, de Lorenzo V, Nikel PI. 2017. Refactoring the Embden-Meyerhof-Parnas Pathway as a Whole of Portable GlucoBricks for Implantation of Glycolytic Modules in Gram-Negative Bacteria. ACS Synth Biol 6:793-805.
