# Supplementary material for: The Inactivation of Enzymes Belonging to the Central Carbon Metabolism Is a Novel Mechanism of Developing Antibiotic Resistance
Source: mSystems. 2020 Jun 2;5(3):e00282-20. doi: 10.1128/mSystems.00282-20 (PMC8534728; doi:10.1128/mSystems.00282-20)
Supplement: TABLE S3 [file msystems.00282-20-st003.docx]

**Table S3. Oligonucleotides** **used in this work**

| **Name** | **Sequence 5´3´** | **Use** |
| --- | --- | --- |
| **Eno_F** | GAATTCATGAGTACGATCCGCAGCATC | Amplification of *eno* EcoRI |
| **Eno_R** | AAGCTTTCAGCGCTTCAGCGAAACGAA | Amplification of *eno* HindIII |
| **GpmA_F** | CGGAATTCTTGGAGACCCTGCAGTGACC | Amplification of *gpmA* EcoRI |
| **GpmA_R** | CCCAAGCTTCTTCTTACTTCGCCTTGCCC | Amplification of *gpmA* HindIII |
| **Pgk_F** | CGGAATTCAGCCAAAGAGTTGCCCATGT | Amplification of *pgk* EcoRI |
| **Pgk_R** | CCCAAGCTTCCTTGCTACGGTCG | Amplification of *pgk* HindIII |
| **GapA_F** | CGGATTCGCAGGAGCTTGAGAAATGGC | Amplification of *gapA* EcoRI |
| **GapA_R** | CCCAAGCTTCGATCTACGCCGGCCTTAC | Amplification of *gapA* HindIII |
| **ZwfAF** | GAATTCGGCTTGCCCGGTACCCAGGCAGCCCCGCCG | Amplification of the 500-bp fragment corresponding to the 5’- end of *zwf* |
| **ZwfAR** | AGGACTTCCGCATCCGTGCGACGCCAAGCTGCCGCTGGAG |  |
| **ZwfBF** | CTCCAGCGGCAGCTTGGCGTCGCACGGATGCGGAAGTCCT | Amplification of the 500-bp fragment corresponding to the 3’- end of *zwf* |
| **ZwfBR** | AATTCGCCGTTGTCATAGACCTTGCGCACACCCTGCAACTGC |  |
| **IntZwf_F** | GCACCGATTCGATGTAGTT | Amplification of a 264-bp fragment inside *zwf* gene |
| **IntZwf_R** | GCCTGTACATCTCCACCTG |  |
| **ExtZwf_F** | ATAGCAACGGCCGTGGCGCAT | Amplification of the 2500-bp fragment including the complete *zwf* gene |
| **ExtZwf_R** | CATCCGCAGCAGGGTCGACTT |  |
| **M13_F** | CACGACGTTGTAAAACGAC | pGEM-T Easy Vector |
| **M13_R** | GGATAACAATTTCACACAGG |  |
| **27**  **48** | TGCCAGCGACAGTGCAAAGGGTC  CCGTGTTCATGGAAGCAGGC | Amplification of *smeT* to test DNA contamination (1) |
| **pSEVA234_F**  **pSEVA234_R** | GCGGATAACAATTTCACACC  AGGGTTTTCCCAGTCACG | Inserts screening in pSEVA234 |
| **227**  **272** | GAACGCTCGGTTGCCGC  AATGACCCCGAAGCAGGG | Specific amplification of *aphA* pSEVA (2) |

1. Sanchez P, Alonso A, Martinez JL. 2002. Cloning and characterization of SmeT, a repressor of the *Stenotrophomonas maltophilia* multidrug efflux pump SmeDEF. Antimicrob Agents Chemother 46:3386-93.

2. Silva-Rocha R, Martinez-Garcia E, Calles B, Chavarria M, Arce-Rodriguez A, de Las Heras A, Paez-Espino AD, Durante-Rodriguez G, Kim J, Nikel PI, Platero R, de Lorenzo V. 2013. The Standard European Vector Architecture (SEVA): a coherent platform for the analysis and deployment of complex prokaryotic phenotypes. Nucleic Acids Res 41:D666-75.
