# Supplementary material for: The Inactivation of Enzymes Belonging to the Central Carbon Metabolism Is a Novel Mechanism of Developing Antibiotic Resistance
Source: mSystems. 2020 Jun 2;5(3):e00282-20. doi: 10.1128/mSystems.00282-20 (PMC8534728; doi:10.1128/mSystems.00282-20)
Supplement: FIG S1 [file msystems.00282-20-sf001.pdf]

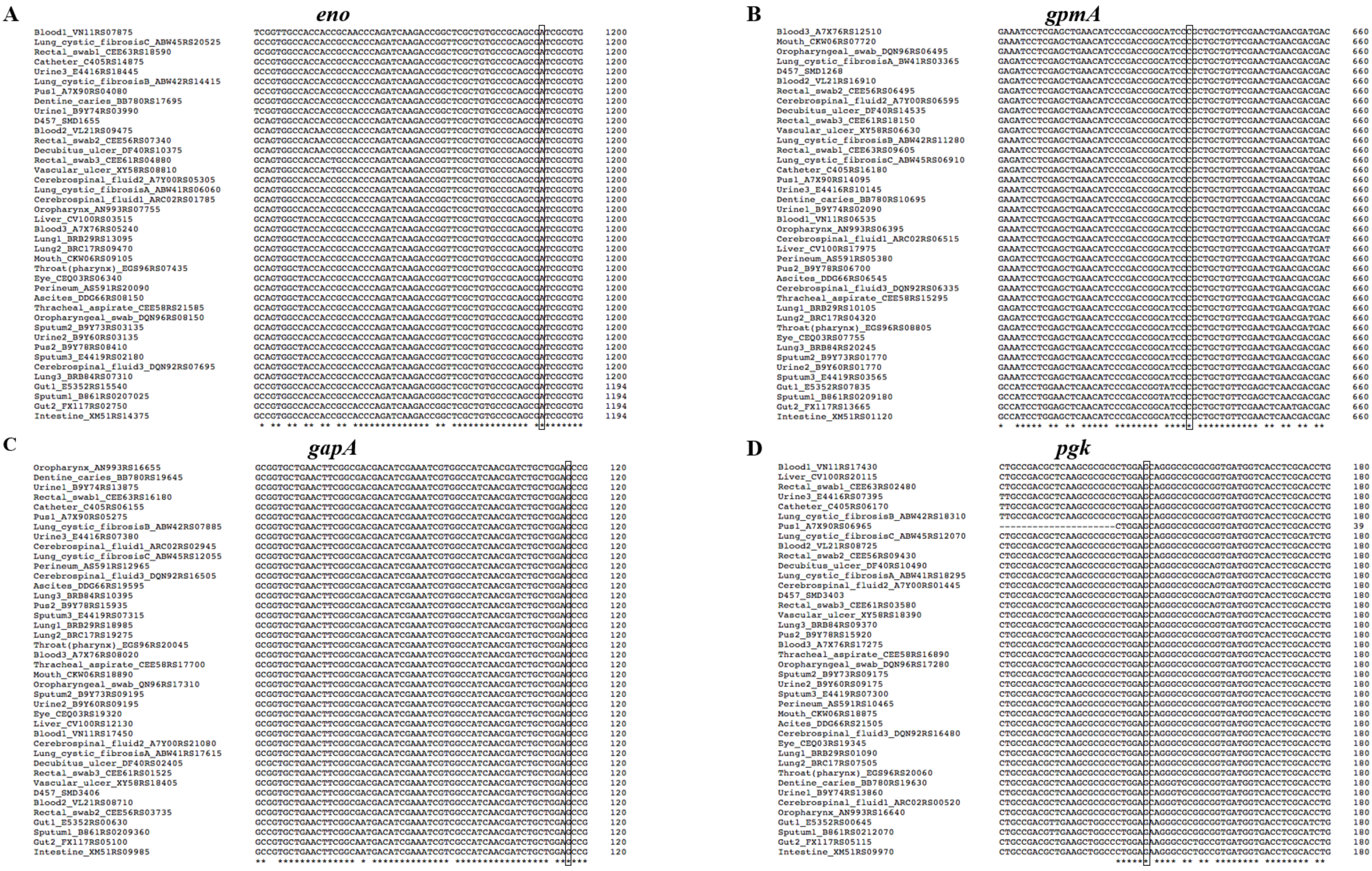

A
eno
B
gpmA
C
gapA
D
pgk
